# Symmetry TFTs from String Theory

**DOI:** 10.1007/s00220-023-04737-2

**Published:** 2023-05-26

**Authors:** Fabio Apruzzi, Federico Bonetti, Iñaki García Etxebarria, Saghar S. Hosseini, Sakura Schäfer-Nameki

**Affiliations:** 1 Albert Einstein Center for Fundamental Physics, Institute for Theoretical Physics, University of Bern, Sidlerstrasse 5, Bern, 3012 Switzerland; 2 Mathematical Institute, University of Oxford, Andrew-Wiles Building, Woodstock Road, Oxford, OX2 6GG UK; 3 Department of Mathematical Sciences, Durham University, Durham, DH1 3LE UK

## Abstract

We determine the $d+1$ dimensional topological field theory, which encodes the higher-form symmetries and their ’t Hooft anomalies for *d*-dimensional QFTs obtained by compactifying M-theory on a non-compact space *X*. The resulting theory, which we call the Symmetry TFT, or SymTFT for short, is derived by reducing the topological sector of 11d supergravity on the boundary $\partial X$ of the space *X*. Central to this endeavour is a reformulation of supergravity in terms of differential cohomology, which allows the inclusion of torsion in cohomology of the space $\partial X$, which in turn gives rise to the background fields for discrete (in particular higher-form) symmetries. We apply this framework to 7d super-Yang Mills, where $X= \mathbb {C}^2/\Gamma _{ADE}$, as well as the Sasaki–Einstein links of Calabi–Yau three-fold cones that give rise to 5d superconformal field theories. This M-theory analysis is complemented with a IIB 5-brane web approach, where we derive the SymTFTs from the asymptotics of the 5-brane webs. Our methods apply to both Lagrangian and non-Lagrangian theories, and allow for many generalisations.

## Introduction

### Symmetry TFTs for QFTs from supergravity

Quantum Field Theories (QFTs) have a rich structure of symmetries: in addition to the familiar symmetry groups acting on point operators one encounters in textbooks, we can also have more general kinds of symmetries: higher-form symmetries [1], higher group symmetries [2–6], non-invertible symmetries [7–12], and more generally symmetries described by abstract categorical structures [13]. Furthermore, theories with identical local dynamics can have different symmetries in this generalised sense [14, 15], and different symmetry structures for a given set of local dynamics can sometimes be related by gauging [16], which in the presence of mixed ’t Hooft anomalies can relate theories with more conventional symmetry structures to theories with less familiar ones [3, 7].

A very useful way of organising these structures, that as we will see seems to arise naturally in string theory, is in terms of the following construction (which we learned from Dan Freed [17], here we are only sketching an outline of a more detailed construction): if the original theory $\mathfrak {T}$ is formulated on *d* dimensional spacetimes $\mathcal {M}_d$, we introduce a generically non-invertible topological $(d+1)$ dimensional quantum field theory (which in this paper we will call the *symmetry theory*, or *symmetry TFT (SymTFT)* when we want to emphasize that it is topological^1^) with the property that it admits a non-topological theory $\tilde{\mathfrak {T}}$ as the theory of edge modes on manifolds with boundary (a relative theory, in the framework of [18]), and also a gapped interface $\rho$ to the anomaly theory $\mathcal {A}$ of the theory $\mathfrak {T}$:
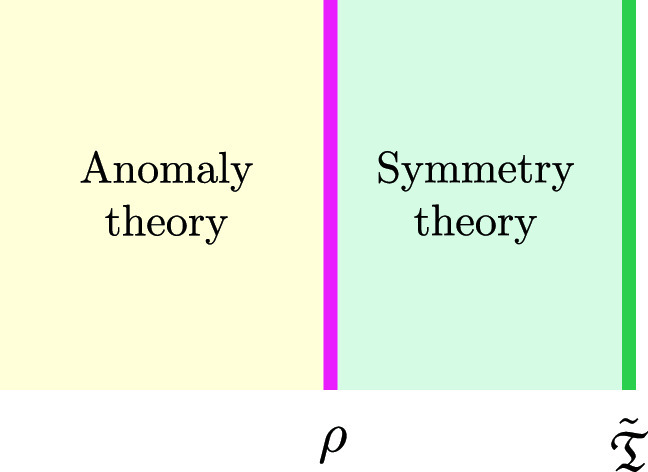


The anomaly theory is a well understood object (see [19, 20] for reviews): it is an invertible theory that gives us a way of defining the phase of the partition function of $\mathfrak {T}$ by evaluating the partition function of $\mathcal {A}$ on a $d+1$ manifold with boundary (see [21] for the original discussion in the case of anomalies of fermions). The theory $\mathfrak {T}$, attached to its anomaly theory $\mathcal {A}$, arises when we collide $\rho$ with $\tilde{\mathfrak {T}}$.

We will argue in this paper that the picture that arises in string theory is the complementary one, in which we focus on the symmetry theory by sending $\rho$ to infinity. More concretely, in this paper we will consider singular string configurations, where we have a set of local degrees of freedom (often strongly coupled) living at the singular point of some non-compact cone *X*. We identify these local degrees of freedom with $\tilde{\mathfrak {T}}$. The choice of the actual symmetries of $\mathfrak {T}$ (which in our picture above would be associated with a choice of $\rho$), has been previously argued to live “at the boundary of *X*” [22–24], a behaviour that is also familiar in the context of holography [25]. The goal of this paper is to sharpen this picture by giving a direct derivation of the symmetry theory from the string construction: we will see that we can obtain in a natural way a non-invertible topological theory encoding both the choices of symmetries for $\mathfrak {T}$ and their anomalies.

Our methods do not require knowledge of a holographic dual, or of a weakly coupled description of the QFT. We find our results particularly illuminating in the case that the local degrees of freedom $\tilde{\mathfrak {T}}$ are those of a strongly coupled CFT without a Lagrangian description (generically we know little about such theories, so any additional information is useful), but we do not require conformality of $\mathfrak {T}$ either.

**Fig. 1:**
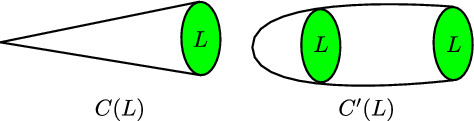
The cone *C*(*L*) over the link *L*, and the deformation, shown on the right, to a long cylinder where the singularity is at the far end

The main idea is as follows. In string theory we can construct the *d*-dimensional theories $\mathfrak {T}$ by introducing defects or singularities extending along *d*-dimensional submanifolds of the 11-dimensional spacetime. For concreteness we will focus in this paper on M-theory on singular spaces with a single isolated singularity. The non-trivial local dynamics arise from massless M2 branes wrapping vanishing cycles at the singularity. Close to the singular point the geometry will look like a real cone over some manifold *L* with $\dim (L)=10-d$. We can deform the cone into an infinitely long cigar, with the singularity at the tip, and *L* as the base of the cylinder along the cigar, see Fig. 1. The information that we are after is topological, so it is reasonable to expect that we can still obtain it from this deformed background (our results will support this expectation). If we now dimensionally reduce the M-theory action on *L* we will obtain a theory on the remaining $d+1$ dimensions, which look like $\mathcal {M}_d\times \mathbb {R}_{\ge 0}$. We claim that the topological sector—i.e. couplings that are metric independent—arising from this reduction on *L* is precisely the symmetry theory for $\tilde{\mathfrak {T}}$.

In our specific context of M-theory we will obtain this topological sector by “reducing” the Chern–Simons sector of M-theory on *L*, additionally including the effect of flux-noncommutativity [26–28]. As we will see, flux-noncommutativity leads to choices of higher form symmetries which appear in a way familiar from holography. For example, in the $\text {AdS}_5 \times S^5$ case studied in [25, 29, 30] the 5d supergravity contains the coupling

1 $$\begin{aligned} S_{\text {Sym}}= {N} \int B_2 \wedge dC_2 , \end{aligned}$$

which upon imposing boundary conditions on $(B_2, C_2)$ yields different global forms of the gauge group of 4d $\mathcal {N}=4$ SYM. (Similar couplings have been studied in other holographic setups such as ABJM in [31] and the non-conformal dual to confinement in Klebanov-Strassler in [32].) We will obtain analogous couplings from compactification. The topological reduction that we consider also generates in a natural way the anomalies that are expected in cases where the answer from field theory is known.

We expect the general idea that we are putting forward to be much more general and applicable in a wide range of setups.^2^ In order to illustrate this point, in Sect. 5 we will apply the same topological reduction prescription for the same 5d SCFTs we analyse from the M-theory viewpoint, but now in terms of their realization from (*p*, *q*) 5-branes [33]. We will show how 1-form symmetries for 5d SCFTs are encoded in the brane-web and compute from a supergravity point of view, expanding on the boundary of the IIB spacetime. The latter is inspired by the back-reacted holographic solutions [34]. These are $\hbox {AdS}_6 \times M_4$ solutions, which are near-horizon limit of (*p*, *q*) 5-branes webs. What will be important for our analysis is the topology of $M_4$ of the near-horizon geometry, which we will be using to dimensionally reduce the topological coupling of IIB supergravity. The topology of $M_4$ is based on isometries as well as asymptotic charges of the semi-infinite 5-branes and it is not affected by any large (*p*, *q*) charge holographic limit, therefore will be valid in any IIB (*p*, *q*) 5-brane-setup. Our focus here is on the dimensional reduction of the IIB 10-dimensional topological coupling on $M_4$ where we also need to include contributions coming from (*p*, *q*) 5-brane source to determine the anomalies for 5d SCFTs for both Lagrangian and non-Lagrangian theories. This beautifully complements the geometric analysis in M-theory. At last, as another application we also derive a mixed anomaly between continuous [6] and discrete 1-form symmetries of the (1, 1) little string theory (LST) engineered by NS5-branes in IIB [35] from the holographic linear dilaton background [36].

### A differential cohomology refinement of dimensional reduction

M-theory compactification on singular Calabi–Yau 2- and 3-folds gives rise to 7d super-Yang Mills (SYM) and 5d superconformal field theories (SCFTs), respectively. These theories have 1-form symmetries, and in the 5d case also 0-form symmetries. The 1-form symmetry in all these cases is discrete and is characterized in terms of the relative homology quotient of the Calabi–Yau *X*, with respect to its boundary $\partial X$ [22–24, 37]

2 $$\begin{aligned} \Gamma ^{(1)}= {H_2 (X, \partial X; \mathbb {Z})\over H_2 (X; \mathbb {Z})}. \end{aligned}$$

To derive SymTFTs for global 1-form symmetries, one will have to incorporate their backgrounds $B_2 \in H^2 (M_5; \Gamma ^{(1)})$ into the supergravity formalism. The torsional nature of these fields introduces various subtleties in the process. We will use differential cohomology to address these subtleties.^3^

Differential cohomology has seen numerous applications within quantum field theory and string/M-theory. Some of the earlier works on the subject include [26, 28, 40–42]. For more recent examples of differential cohomology applications in formal high-energy physics that are of some relevance to this work see [16, 43–52]. In this paper differential cohomology will be used to refine the notion of dimensional reduction (or KK-reduction) of supergravity theories, with the goal of providing a precise treatment of the effect of torsion cohomology classes in the compactification manifold.

To illustrate, without going into the mathematical intricacies of differential cohomology yet, we now give a very concrete example of how the concept of dimensional reduction is reformulated using this approach. We begin with M-theory on a 5d space $L_5$ (which in this paper will be a manifold linking the singular point of a non-compat Calabi–Yau three-fold) which has $H^2 (L_5; \mathbb {Z}) = \mathbb {Z}_n \oplus \mathbb {Z} = \langle t_2 \rangle \oplus \langle v_2\rangle$, where $t_2$ is a torsional generator of the degree two cohomology group and $v_2$ is a free generator. The reduction for the latter is the standard KK-reduction. It is the torsion part that will most benefit from the uplift to differential cohomology. We denote the differential cohomological uplifts of $t_2$ and $v_2$ by $\breve{t}_2$ and $\breve{v}_2$, the precise meaning of these will be explained below.

In the case of M-theory we need to introduce a differential refinement $\breve{G}_4$ of $G_4$ as well. As in the standard KK-expansion, this has a decomposition in terms of differential cohomology classes along the internal space $L_5$ (torsion and free), as well as external spacetime $M_6$:

3 $$\begin{aligned} \breve{G}_4 = \breve{F} \star \breve{v}_2 + \breve{B}_2 \star \breve{t}_2. \end{aligned}$$

There is an extra term, discussed below, that we are ignoring here for simplicity. The meaning and properties of the product “$\star$” will also be explained below. The CS-term in the M-theory action is

4 $$\begin{aligned} \frac{S_{\text {top}}}{2\pi }=-\frac{1}{6} \int _{M_{11}} \breve{G}_4 \star \breve{G}_4 \star \breve{G}_4 , \end{aligned}$$

which upon inserting the decomposition (3) of $\breve{G}_4$ we integrate over $L_5 \times \mathcal {W}_6$. The integration over the internal space $L_5$ results in the SymTFT on $\mathcal {W}_6$. In ordinary cohomology the integral on $L_5$ would pick out only the forms of degree 5, however that would mean the purely torsional part $\int _{L_5} \breve{t}_2 \star \breve{t}_2 \star \breve{t}_2$ would naively not contribute. Differential cohomology works differently, and as reviewed below $\int _{L_5} \breve{t}_2 \star \breve{t}_2 \star \breve{t}_2$ can be non-vanishing.

Reformulating the problem in terms of differential cohomology on the link of the singularity involves some additional technical complications, but the effort pays off in a number of ways:

- Geometric engineering of QFTs corresponds to “compactification” of string and M-theory on non-compact spaces $Y^d$. This can be mathematically challenging, in particular it is difficult to define in a precise mathematical sense what one means by reducing the Chern–Simons action on a non-compact space $Y^d$. In our approach we are instead reducing the Chern–Simons action on the closed manifold $X^{d-1}= \partial Y^d$, which is the boundary or link of the non-compact space $Y^d$. This is a much better defined mathematical question, that can be clearly analysed using the formalism of differential cohomology.
- The effective field theory in $11-d$ dimensions is most interesting when $Y^{d}$ is singular, so it becomes a non-abelian Yang-Mills theory in 7d (for $d=4$) or a non-trivial interacting 5d SCFT (for $d=6$). But it is precisely in this singular geometric regime that it is most difficult to pin down what one means by doing a geometric reduction of the effective action. By contract, our formalism is entirely agnostic about the singular structure of $Y^d$, and can be applied without issues even when $Y^d$ is singular (in fact, it is arguably at the singular cone point in moduli space where it is most natural to apply our techniques!).
- Often, the analysis of reduction on singular spaces is done by removing these singularities, e.g. in 5d going to the Coulomb branch. It is well-documented, e.g. in the set of canonical singularities realizing 5d SCFTs from isolated hypersurface singularities (see [53–56] for a discussion in the context of 5d SCFTs), that we can have terminal singularities that do not admit a Calabi–Yau (crepant) resolution. This is obviously a class of theories where many standard methods will fail. Another setting where field theory-inspired arguments (including those employed in [57]) are not extendable, is when the 5d SCFT may have a Coulomb branch, but does not admit a non-abelian gauge theory description. In contrast, our approach of deriving the SymTFT and thereby the anomaly of the QFT in terms of the reduction on the boundary is applicable in all those instances, and we will provide some examples of non-Lagrangian 5d SCFTs and their SymTFTs below.
- The approach uniformly encodes the entire SymTFT, for all symmetries arising from the compactification. E.g. in 5d we derive both the mixed 0–1-form symmetry anomalies as well as the 1-form symmetry $(B_2)^3$ anomaly [58] (see Sect. 4).
- This approach lends itself to applications also in the context of string and M-theory compactifications with torsional cycles are in the geometry, such as those studied in [38, 39], which give rise to theories with discrete gauge symmetries in string theory.

### Comparison to other approaches

Continuous global symmetries are usually rather manifest in terms of the geometry, e.g. the R-symmetry of a 4d $\mathcal {N}=1$ SCFT is often encoded in some geometric isometry (such as in the setup of D3-branes probing Calabi–Yau cones), or the flavor symmetry in terms of non-compact divisors in a Calabi–Yau compactification—as will be the case in this paper. Discrete and continuous higher-form symmetries are encoded also in the topology of the compactification space—usually in terms of relative homology, that captures the defect operators, modulo screening [59]. In the reduction on the link, the anomalies arise in terms of topological couplings for the background fields of global symmetries. Systematic tools for treating symmetries associated to isometries and non-torsional cohomology classes in brane constructions in M-theory and Type IIB are studied in [60, 61] in connection to equivariant cohomology, see also [62].

In this paper we focus instead mostly on the torsional sector. Dealing with torsion cycles in supergravity has of course a history. One particularly promising framework was put forward in [38, 39]. In supergravity, form-fields are usually expanded in harmonic forms, which however do not capture the torsion parts $\textrm{Tor }\, H^p(X_d;{\mathbb {Z}})$. The key idea of these papers is to use non-harmonic forms to model classes in $\textrm{Tor }\, H^p(X_d;{\mathbb {Z}})$. More precisely, a non-trivial class in $\textrm{Tor }\, H^p(X_d,{\mathbb {Z}})$ of order *k* is modeled by a $(p-1)$-form $\beta _{p-1}$ and a *p*-form $\alpha _{p}$ subject to the condition

5 $$\begin{aligned} k \alpha _p = d\beta _{p-1}. \end{aligned}$$

Moreover, motivated by the above general remarks on the KK-expansion, $\beta _{p-1}$ is required to be a co-exact eigenfunction of the Laplacian with a non-zero eigenvalue. (It then follows automatically that $\alpha _p$ is also an eigenform with the same eigenvalue.)

To illustrate the physical picture underlying this proposal, let us consider for example the terms in the expansion of $C_3$ associated to a pair $(\beta _1, \alpha _2)$ satisfying (5). We have schematically

6 $$\begin{aligned} C_3 \supset {\widetilde{B}}_2 \wedge \beta _1 + {\widetilde{A}}_1 \wedge \alpha _2 \ , \qquad G_4 \supset (d{\widetilde{A}}_1 + k {\widetilde{B}}_2) \wedge \alpha _2 + d{\widetilde{B}}_2 \wedge \beta _1 . \end{aligned}$$

The 11d kinetic term for $G_4$ induces lower-dimensional kinetic terms of the schematic form

7 $$\begin{aligned} S \supset \int - \frac{1}{2} \, g_{{\widetilde{A}}_1 {\widetilde{A}}_1} \, (d{\widetilde{A}}_1 + k {\widetilde{B}}_2) \wedge * (d{\widetilde{A}}_1 + k {\widetilde{B}}_2) - \frac{1}{2} \, g_{{\widetilde{B}}_2 {\widetilde{B}}_2} \, d {\widetilde{B}}_2 \wedge * d {\widetilde{B}}_2 \ , \end{aligned}$$

where the Hodge star and the integration are now over external spacetime in $11-d$ dimensions. The quantities $g_{{\widetilde{A}}_1 {\widetilde{A}}_1}$, $g_{{\widetilde{B}}_2 {\widetilde{B}}_2}$ depend on the details of the compactification and on the eigenvalue of the forms $\beta _1$, $\alpha _2$ under the action of the internal Laplacian. We do not need to discuss them in detail for this argument. We observe that the lower-dimensional fields ${\widetilde{A}}_1$, ${\widetilde{B}}_2$ participate in a Stückelberg mechanism: the 2-form field ${\widetilde{B}}_2$ “eats” the 1-form field ${\widetilde{A}}_1$ and gets massive (as is expected, since the eigenforms $\beta _1, \alpha _2$ have a non-zero eigenvalue of the internal Laplacian). The Stückelberg mechanism leaves behind an unbroken ${\mathbb {Z}}_k$ gauge symmetry: in the IR, the field ${\widetilde{B}}_2$ is a continuum description of a ${\mathbb {Z}}_k$ discrete 2-form gauge field. We identify the latter with the discrete gauge field that originates from a formal expansion of $G_4$ of the form $G_4 \supset {\widetilde{B}}_2 \cup x$, where $x\in \textrm{Tor} \, H^2(X_d;{\mathbb {Z}})$ is the torsion class modelled by the pair of forms $(\beta _1,\alpha _2)$.

This approach has been applied successfully in various compactification scenarios with torsion (co)-cycles. However, clearly, this prescription leaves various mathematical questions open, and equally importantly, in more complicated compactification settings, carrying out this approach consistently can be difficult. The differential cohomology approach that we propose here has the advantage of providing a sound mathematical framework, which unambiguously lets us implement torsion in a supergravity setting. It would be interesting to provide a precise map to the above prescription using non-closed forms.

We should also comment that the M-theory approach often has a IIA-avatar, e.g. when there is a circle-fibration in the geometry. We will see that this can be a useful complementary check of our proposal. However, obviously this only applies in a very limited set of geometric situations. To contrast and compare to the IIA setting, we discuss the IIA counterparts to the M-theory computations in this paper in Appendix B.

In the context of 5d SCFTs and 7d gauge theories based on elliptic Calabi–Yau singularities, the paper [57] has discussed in specific instances^4^ the anomalies of 1-form symmetries. The analysis is based on a Coulomb branch (CB) computation in the resolved geometry. What is implemented is a combination of a field theoretic analysis, with information from the geometry about the structure of the CB. The point of view in that paper is roughly complementary to ours: they integrate the Chern–Simons term on the singular Calabi–Yau $X_d$ to obtain an anomalous coupling in the $(11-d)$-dimensional field theory, while we integrate the Chern–Simons coupling on the $(d-1)$-dimensional link of the singular point in $X_d$ to obtain the $(10-d)$-dimensional anomaly theory (together with the non-invertible sectors, which are not considered in [57]). As emphasised above, this change in perspective has many benefits, for instance it allows us to approach problems where we do not have a gauge theory description to guide us.

We also resolve a puzzle in the computation in [57] of the mixed anomaly between the 1-form symmetry and the instanton symmetry. It was observed in that paper that the results of the computation did not always agree with the field theory expectation [63]. In the present paper, we obtain the expected field theory mixed anomaly,^5^ and in addition also compute the $B^3$ 1-form symmetry anomaly, where we also find agreement with [58]. Reproducing these very involved results gives us confidence that our approach is both sound and fruitful. More generally we expect to be able to apply our approach to non-geometric, and also not-resolvable geometries, which would provide a substantial extension compared to both field theory and Coulomb branch approaches.

***Plan.***

The plan of this paper is as follows: We start in Sect. 2 with an overview of differential cohomology, providing a (hopefully physics-friendly) summary of its salient features. We then apply this to the M-theory topological couplings—the CS and $C_3\wedge X_8$ terms, and give the general KK-expansion in terms of differential cohomology. This framework is put to work in the context of $\mathbb {C}^2/\Gamma _{ADE}$ compactifications in M-theory in Sect. 3 and in Sect. 4 to 5d SCFTs realized on canonical singularities in Calabi-Yau- three-folds. The anomalies of the 1-form symmetry are determined in these cases, including the mixed 0–1-form symmetry anomaly in 5d. For 5d we derive the anomalies from a complementary point of view in Sect. 5 using the 5-brane webs in Type IIB—again from the boundary of the spacetime. Finally, we determine the anomaly of the little string theory (LST) from an edge mode approach in Sect. 6. In Appendix A we address a technical point regarding $G_4$-flux quantisation and in Appendix B we provide a countercheck to the M-theory results, using more conventional IIA reductions, which are available in some setups.

## Differential Cohomology and M-Theory

In this section we discuss how to apply the language of differential cohomology to describe the topological couplings in the M-theory low-energy effective action. We then describe the dimensional reduction of these couplings on a generic internal space *L* including contributions originating from both free and torsion elements in the cohomology of *L*.

### Aspects of differential cohomology

Differential cohomology provides a mathematical framework to describe *U*(1) $(p-1)$-form gauge fields on arbitrary spacetimes. This formalism is particularly useful to keep track of subtler aspects that emerge when we consider spacetimes with non-trivial topology (and in particular with torsional cycles). We refer the reader to [27, 28, 49, 64–66] for reviews aimed at physicists. We also highly recommend the textbook by Bär and Becker [67] for a pedagogical discussion of most of the results below.

***Characteristic class and field strength.***

Let us consider a closed, connected, oriented manifold $\mathcal {M}$. The *p*-th differential cohomology group $\breve{H}^p(\mathcal {M})$ of $\mathcal {M}$ is an Abelian group furnishing a differential refinement of the ordinary cohomology group $H^p(\mathcal {M};{\mathbb {Z}})$. The group $\breve{H}^p(\mathcal {M})$ sits at the center of the following commutative diagram: 
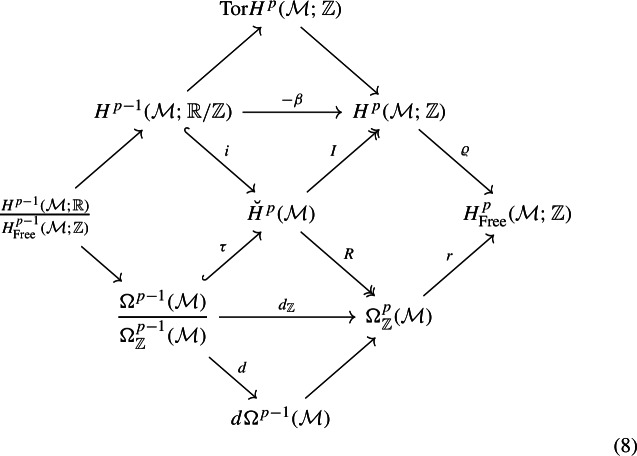
 where all the diagonals are short exact sequences.

The maps $i, I, \tau , R$ are natural (that is, given a smooth map $f:\mathcal {M}\rightarrow \mathcal {M}'$ they commute with the pullback $f^*$ of *f*). Let us now proceed to unpack the relevant information contained in the above diagram, and to provide some physical interpretation:

- The symbol $\Omega _{\mathbb {Z}}^q(\mathcal {M})$ denotes closed differential *q*-forms on $\mathcal {M}$ with integral periods. The surjective map *R* associates to each element $\breve{a} \in \breve{H}^p(\mathcal {M})$ a *p*-form $R(\breve{a}) \in \Omega ^p_{{\mathbb {Z}}}(\mathcal {M})$, which we refer to as the field strength of $\breve{a}$. Physically, $\breve{a}$ models a *U*(1) $(p-1)$-form field, up to gauge equivalences, and the *p*-form $R(\breve{a})$ is identified with the physical field strength of the $(p-1)$-form gauge field. (The fact that $R(\breve{a})$ has integral periods encodes the fact that the gauge group is *U*(1) and not ${\mathbb {R}}$.)
- An element $\breve{a} \in \breve{H}^p(\mathcal {M})$ with $R(\breve{a}) = 0$ is called flat. Exactness of the central NW-SE diagonal in the diagram (8) demonstrates that flat elements of $\breve{H}^p(\mathcal {M})$ can be identified with elements in $H^{p-1}(\mathcal {M}; {\mathbb {R}}/\mathbb Z)$. Physically, the gauge-invariant information about a flat $(p-1)$-form gauge field is encoded in its holonomies around non-trivial $(p-1)$-cycles, which take values in $U(1) \cong \mathbb R/{\mathbb {Z}}$ and can be encoded in an element of $H^{p-1}(\mathcal {M}; {\mathbb {R}}/{\mathbb {Z}})$.
- The surjective map $I: \breve{H}^p(\mathcal {M}) \rightarrow H^p(\mathcal {M}; {\mathbb {Z}})$ is the map that “forgets” the differential refinement, yielding back ordinary cohomology with coefficients in ${\mathbb {Z}}$. Given an element $\breve{a} \in \breve{H}^p(\mathcal {M})$, we refer to $I (\breve{a}) \in H^p(\mathcal {M};{\mathbb {Z}})$ as the characteristic class of $\breve{a}$.
- An element $\breve{a} \in \breve{H}^p(\mathcal {M})$ with $I(\breve{a}) = 0$ is called topologically trivial. Exactness of the central SW-NE diagonal in the diagram (8) implies that topologically trivial elements of $\breve{H}^p(\mathcal {M})$ can be identified with globally defined $(p-1)$-forms on $\mathcal {M}$, up to additive shifts by closed $(p-1)$-forms with integral periods. In physics term, a topologically trivial $(p-1)$-form gauge field can be described globally by specifying a $(p-1)$-form. The shift by closed $(p-1)$-forms with integral periods is interpreted as a gauge transformation (a “large gauge transformation” if the $(p-1)$-form is closed but not exact).
- Commutativity of the square on the RHS of the diagram (8) is the statement that, for any $\breve{a} \in \breve{H}^p(\mathcal {M})$,
  9 $$\begin{aligned} r\big ( R(\breve{a}) \big ) = \varrho \big ( I(\breve{a}) \big ) . \end{aligned}$$
  The short exact sequence in the lower NW-SE diagonal of (8) comes from the isomorphism $\Omega ^{p}_\mathbb {Z}(\mathcal {M})/d\Omega ^{p-1}(\mathcal {M})\cong H_{\text {free}}^p(\mathcal {M};\mathbb {Z})$ which is a by-product of de Rham’s theorem. From the physics perspective, it is well-known that information about the topological aspects of a $(p-1)$-form gauge field configuration can be extracted from its field strength (for example, the integer charge of a monopole configuration for a *U*(1) 1-form gauge field on $\mathcal {M}= S^2$ is extracted integrating the 2-form field strength on $S^2$). Crucially, however, the field strength encodes only $\varrho (I(\breve{a}))$ and not necessarily $I(\breve{a})$. To see this, let $I(\breve{a})=[a]\in H^p(\mathcal {M}; \mathbb {Z})$ and embed ${\mathbb {Z}}$ into ${\mathbb {R}}$ to get $[a]_{{\mathbb {R}}}\in H^p(\mathcal {M}; {\mathbb {R}})$. Then, for a de Rham cohomology class $[F]_{\text {dR}}\in H_{\text {Free}}^p(\mathcal {M}; \mathbb Z)\otimes {\mathbb {R}}$ of $F\in \Omega ^p_{{\mathbb {Z}}}(\mathcal {M})$ we have $[F]_{\text {dR}}=[a]_{{\mathbb {R}}}$. Thus, [*a*] contains more information than $[F]_{\text {dR}}$ at the differential level since $[a]_{{\mathbb {R}}}$ can be obtained from [*a*] but the converse is not true. In particular, information about torsional components in $I( \breve{a} )$ is lost in passing to $\varrho (I(\breve{a}))$.
- A flat element in $\breve{H}^p(\mathcal {M})$ is not necessarily topologically trivial. Suppose $\breve{a} \in \breve{H}^p(\mathcal {M})$ is flat; we aim to compute its characteristic class $I(\breve{a})$. From exactness of the NE-SW diagonal we know that $\breve{a} = i(u)$ for some $u \in H^{p-1}(\mathcal {M}; {\mathbb {R}}/{\mathbb {Z}})$. Commutativity of the upper triangle in the diagram (8) gives us
  10 $$\begin{aligned} I(\breve{a} ) = I (i(u)) = - \beta (u) . \end{aligned}$$
  Here $\beta : H^{p-1}(\mathcal {M}; {\mathbb {R}}/{\mathbb {Z}}) \rightarrow H^p(\mathcal {M}; {\mathbb {Z}})$ is the Bockstein homomorphism associated to the short exact sequence $0 \rightarrow {\mathbb {Z}} \rightarrow {\mathbb {R}} \rightarrow {\mathbb {R}} / {\mathbb {Z}} \rightarrow 0$,
  11 $$\begin{aligned}{} & {} \ldots \rightarrow H^{p-1}(\mathcal {M}; \mathbb Z) \xrightarrow {\varrho } H^{p-1}(\mathcal {M};\mathbb {R}) \rightarrow H^{p-1}(\mathcal {M}; {\mathbb {R}} /{\mathbb {Z}})\nonumber \\{} & {} \quad \xrightarrow {\beta } H^p(\mathcal {M}; {\mathbb {Z}} ) \xrightarrow {\varrho } H^p(\mathcal {M}; {\mathbb {R}} ) \rightarrow \ldots \end{aligned}$$
  which is in general non-vanishing.
- A topologically trivial element in $\breve{H}^p(\mathcal {M})$ is not necessarily flat. Suppose $\breve{a} \in \breve{H}^p(\mathcal {M})$ is topologically trivial; we aim to compute its field strength $R(\breve{a})$. From exactness of the central SW-NE diagonal we know that $\breve{a} = \tau ([\omega ])$ for some class $[\omega ]$ in the quotient $\Omega ^{p-1}(\mathcal {M}) / \Omega ^{p-1}_{\mathbb {Z}}(\mathcal {M})$. Commutativity of the lower triangle in the diagram (8) gives us
  12 $$\begin{aligned} R(\breve{a} ) = R (\tau ([\omega ])) = d_{{\mathbb {Z}}}[\omega ] . \end{aligned}$$
  The symbol $d_{{\mathbb {Z}}}$ in the diagram denotes the standard de Rham differential on forms, which passes to the quotient of $\Omega ^{p-1}(\mathcal {M})$ by $\Omega ^{p-1}_{\mathbb {Z}}(\mathcal {M})$. The relation (12) is familiar in physics: if we have a topologically trivial $(p-1)$-form gauge field, described by the globally defined form $\omega$ in some gauge, its field strength is simply $d\omega$.
- An element $\breve{a} \in \breve{H}^p(\mathcal {M})$ can be both flat and topologically trivial. Such elements in $\breve{H}^p(\mathcal {M})$ are usually referred to as Wilson lines. A Wilson line in $\breve{H}^p(\mathcal {M})$ can be identified with an element in the quotient $H^{p-1}(\mathcal {M};\mathbb R)/H^{p-1}_{\text {Free}}(\mathcal {M};{\mathbb {Z}})\cong H^{p-1}(\mathcal {M};\mathbb Z)\otimes \mathbb {R}/ \mathbb {Z}$. The latter is in turn isomorphic to
  13 $$\begin{aligned} \frac{H^{p-1}(\mathcal {M};{\mathbb {R}})}{H^{p-1}_{\text {Free}}(\mathcal {M};{\mathbb {Z}})} \cong \frac{\Omega _{\textrm{closed}}^{p-1}(\mathcal {M})}{ \Omega ^{p-1}_\mathbb Z(\mathcal {M})} , \end{aligned}$$
  which is a torus of dimension $b^{p-1} = \dim H^{p-1}(\mathcal {M};\mathbb R)$.

Two differential cohomology classes $\breve{a},\breve{b}\in \breve{H}^p(\mathcal {M})$ with $I(\breve{a})=I(\breve{b})$ necessarily differ by a topologically trivial class. Exactness of the central NW-SE exact sequence in (8) then implies that $\breve{a} - \breve{b}$ can be represented by an element in $\Omega ^{p-1}(\mathcal {M})/\Omega ^{p-1}_\mathbb {Z}(\mathcal {M})$. We conclude that we can view $\breve{H}^p(\mathcal {M})$ as a fibration with basis the set of points in $H^p(\mathcal {M};\mathbb {Z})$, and fiber isomorphic to $\Omega ^{p-1}(\mathcal {M})/\Omega ^{p-1}_\mathbb {Z}(\mathcal {M})$:14Concretely, if we pick some origin $\breve{\Phi }$ for the fiber on top of $I(\breve{\Phi })$, we can write the most general element $\breve{a}$ of the fiber as

15 $$\begin{aligned} \breve{a} = \breve{\Phi }+ \tau ([\omega ]) \ , \end{aligned}$$

where $\omega \in \Omega ^{p-1}(X)$ is a differential form representing a class $[\omega ]$ in the quotient of $\Omega ^{p-1}(\mathcal {M})$ by $\Omega ^{p-1}_\mathbb {Z}(\mathcal {M})$. As pointed out above, a different choice for $\omega$ in the same class $[\omega ]$ is simply a gauge transformation.

***Torsion Classes.***

Let us consider a torsion cohomology class $t\in H^p(\mathcal {M}; \mathbb Z)$. It will be useful for us to choose a convenient origin $\breve{\Phi }$ for the fiber on top of *t*. By exactness of the long exact sequence (11), we have that if $t\in H^{p}(\mathcal {M};\mathbb {Z})$ is torsion then there is some (not necessarily unique) $u\in H^{p-1}(\mathcal {M}; {\mathbb {R}}/{\mathbb {Z}})$ such that $t = - \beta (u)$. Our choice for the origin of the fiber above *t* is $\breve{\Phi }= i(u)$. Commutativity of (8) ensures $I(\breve{\Phi }) = t$, confirming indeed that $\breve{\Phi }$ lies in the fiber on top of *t*. Moreover, the differential cohomology class $\breve{\Phi }$ is flat, $R(\breve{\Phi }) = 0$, as follows from exactness of the central NW-SE diagonal in (8).

***Product structure in differential cohomology.***

There exists a bilinear product operation on differential cohomology classes,

16 $$\begin{aligned} \star :\quad \breve{H}^p(\mathcal {M})\times \breve{H}^q(\mathcal {M})\rightarrow \breve{H}^{p+q}(\mathcal {M}) . \end{aligned}$$

The product $\star$ is natural and satisfies the following identities: for any $\breve{a} \in \breve{H}^p(\mathcal {M}),\breve{b} \in \breve{H}^q(\mathcal {M})$,

17 $$\begin{aligned} \breve{a} \star \breve{b} = (-)^{pq} \, \breve{b} \star \breve{a} \ , \qquad I(\breve{a} \star \breve{b}) = I(\breve{a}) \smile I(\breve{b}) \ , \qquad R(\breve{a} \star \breve{b}) = R(\breve{a}) \wedge R(\breve{b}) . \end{aligned}$$

In the above relations, $\wedge$ is the standard wedge product of differential forms and $\smile$ is the standard cup product of cohomology classes.

The product of a topologically trivial (respectively flat) element in $\breve{H}^p(\mathcal {M})$ with any element in $\breve{H}^q(\mathcal {M})$ is again topologically trivial (respectively flat). More precisely, we have the identities

18 $$\begin{aligned} \tau ([\omega ]) \star \breve{b} = \tau ([ \omega \wedge R(\breve{b}) ]) \ , \qquad i(u) \star \breve{b} = i ( u \smile I(\breve{b})) \ , \end{aligned}$$

for any $\omega \in \Omega ^{p-1}(\mathcal {M}), u\in H^{p-1}(\mathcal {M}; {\mathbb {R}} /{\mathbb {Z}})$, and $\breve{b} \in \breve{H}^q(\mathcal {M})$.^6^ Recall that $[\omega ]$ denotes the equivalence class of $\omega$ in $\Omega ^{p-1}(\mathcal {M})/\Omega _{\mathbb {Z}}^{p-1}(\mathcal {M})$.

***Fiber integration in differential cohomology.***

Given a locally trivial fiber bundle $\mathcal {M}$ with base $\mathcal {B}$ and closed fiber ${\mathcal {F}}$, we can define an integration over the fiber

19 $$\begin{aligned} \int _{{\mathcal {F}}} :\qquad \breve{H}^p(\mathcal {M}) \rightarrow \breve{H}^{p-\dim ({\mathcal {F}})}(\mathcal {B}) , \end{aligned}$$

which we can characterize axiomatically. First, it is a natural group homomorphism that is compatible with taking the curvature and taking the characteristic class:

20 $$\begin{aligned} \int _{{\mathcal {F}}} R(\breve{a}) = R\left( \int _{{\mathcal {F}}} \breve{a}\right) \, , \qquad \int _{{\mathcal {F}}} I(\breve{a}) = I\left( \int _{{\mathcal {F}}} \breve{a}\right) . \end{aligned}$$

(On the left hand side of these expressions we are using the usual notions of fiber integration of differential forms and cohomology classes.) It is also compatible with the maps *i* and $\tau$:

21 $$\begin{aligned} \int _{{\mathcal {F}}} i(u) = i\left( \int _{{\mathcal {F}}} u\right) , \qquad \int _{{\mathcal {F}}} \tau ([\omega ]) = \tau \left( \left[ \int _{{\mathcal {F}}} \omega \right] \right) . \end{aligned}$$

An important special case is when we take $\mathcal {B}= \textrm{pt}$ and we identify the fiber ${\mathcal {F}}$ with $\mathcal {M}$ itself. One has $\breve{H}^0(\textrm{pt}) \cong {\mathbb {Z}}, \breve{H}^1(\textrm{pt}) \cong {\mathbb {R}} / {\mathbb {Z}}$, while $\breve{H}^p(\textrm{pt})$ is trivial for $p \ne 0,1$. We then have two non-trivial integration maps. The first is integer-values and yields the so-called *primary invariant* of a differential cohomology class of degree $\dim (\mathcal {M})$,

22 $$\begin{aligned} \int _\mathcal {M}\breve{a} = \int _\mathcal {M}I(\breve{a}) = \int _\mathcal {M}R(\breve{a}) \in {\mathbb {Z}} \ , \qquad \breve{a} \in \breve{H}^{\dim (\mathcal {M})}(\mathcal {M}) . \end{aligned}$$

The second integration operator is valued in ${\mathbb {R}} /{\mathbb {Z}}$ and yields the so-called *secondary invariant* of a differential cohomology class of degree $\dim (\mathcal {M})+1$,

23 $$\begin{aligned} \int _\mathcal {M}\breve{a} = \int _\mathcal {M}u \in {\mathbb {R}} /{\mathbb {Z}} \ , \quad \breve{a} \in \breve{H}^{\dim (\mathcal {M}) + 1}(\mathcal {M}) \ , \quad u \in H^{\dim (\mathcal {M})}(\mathcal {M}; {\mathbb {R}}/{\mathbb {Z}}) \ , \quad \breve{a} = i(u) . \end{aligned}$$

We have used the fact that any element $\breve{a} \in \breve{H}^{\dim (\mathcal {M}) +1}(\mathcal {M})$ is necessarily flat for dimensional reasons, and therefore can be written as $\breve{a} = i(u)$ for some $u \in H^{\dim (\mathcal {M})}(\mathcal {M}; {\mathbb {R}}/{\mathbb {Z}})$.

### Differential cohomological formulation of M-theory

The topological terms in the M-theory low-energy effective action can be written schematically in the form

24 $$\begin{aligned} e^{i S_{\textrm{top}}} = \exp \, 2\pi i \, \int _{\mathcal {M}_{11}} \bigg [ - \frac{1}{6} \, C_3 \wedge G_4 \wedge G_4 - C_3 \wedge X_8 \bigg ] \ , \end{aligned}$$

where $\mathcal {M}_{11}$ is 11d spacetime, $C_3$ is the M-theory 3-form gauge field, $G_4$ is its field strength, and $X_8$ is an 8-form characteristic class constructed from the Pontryagin classes $p_i(T\mathcal {M}_{11}), i=1,2$, of the tangent bundle to $\mathcal {M}_{11}$,

25 $$\begin{aligned} X_8 = \frac{1}{192} \, \bigg [ p_1(T\mathcal {M}_{11})^2 - 4 \, p_2(T\mathcal {M}_{11}) \bigg ] . \end{aligned}$$

The expression (24) for the topological couplings can only be taken literally if the 3-form is topologically trivial, in which case $C_3$ is a globally defined 3-form on $\mathcal {M}_{11}$, and the integral in (24) can be understood as the standard integral of an 11-form. In topologically non-trivial situations, such as those studied in this work, greater care is needed to make sense of the formal expression (24).

For our purposes, it will be enough to model the M-theory 3-form gauge field as a class $$\breve{G}_4 \in \breve{H}^4(\mathcal {M}_{11})$$ in (ordinary) differential cohomology.^7^$$^{,}$$^8^ In particular, we are implicitly restricting ourselves to situations in which the periods of the M-theory 4-form field strength are integrally quantized. As explained in [69], on certain spacetimes the periods must be half-integrally quantized. We argue in Appendix A that this does not occur in the setups discussed in this work.^9^

The topological action (24) is interpreted as the ${\mathbb {R}}/{\mathbb {Z}}$-valued secondary invariant of a differential cohomology class $\breve{I}_{12} \in \breve{H}^{12}(\mathcal {M}_{11})$,

26 $$\begin{aligned} \frac{S_{\textrm{top}}}{2\pi } = \int _{\mathcal {M}_{11}} \breve{I}_{12} \mod 1 \ , \end{aligned}$$

where $\breve{I}_{12}$ is given by

27 $$\begin{aligned} \breve{I}_{12} = - \frac{1}{6} \, \breve{G}_4 \star \breve{G}_4 \star \breve{G}_4 - \frac{1}{192} \, \breve{G}_4 \star \breve{p}_1(T\mathcal {M}_{11}) \star \breve{p}_1(T \mathcal {M}_{11}) + \frac{1}{48} \, \breve{G}_4 \star \breve{p}_2(T\mathcal {M}_{11}) . \end{aligned}$$

In the previous expression, $\breve{p}_i(T\mathcal {M}_{11}) \in \breve{H}^{4i}(\mathcal {M}_{11})$ denotes a differential refinement of the Pontryagin classes $p_i(T\mathcal {M}_{11}) \in H^{4i}(\mathcal {M}_{11};{\mathbb {Z}})$ [65, 71, 72].

Within the formalism of differential cohomology we are allowed to consider products and ${\mathbb {Z}}$-linear combinations of differential cohomology classes, but multiplying by rational coefficients—such as the factor of 1/6 in front of the $\breve{G}_4 \star \breve{G}_4 \star \breve{G}_4$ term in (27)—leads to a quantity which is not well defined in general. The fact that the particular combination $\breve{I}_{12}$ is nonetheless well-defined stems from the analysis of [69, 73], which demonstrates that the total topological action $e^{i S_{\textrm{CS}}}$ is well-defined up to a sign, which cancels a potential sign problem in the definition of the Rarita-Schwinger determinant. This sign ambiguity arises if and only if the periods of the $G_4$ field strength are half-integrally quantized. As mentioned above, this does not occur for the setups discussed in this work, meaning that $e^{i S_{\textrm{CS}}}$ is well-defined by itself.

### Kaluza–Klein reduction in differential cohomology

Let us consider an 11d spacetime $\mathcal {M}_{11}$ that is the direct product of an “internal” manifold $L_n$ of dimension *n*, and an “external” spacetime $\mathcal {W}_{11-n}$ of dimension $11-n$,

28 $$\begin{aligned} \mathcal {M}_{11} = \mathcal {W}_{11-n} \times L_n . \end{aligned}$$

It is standard to consider the expansion of the M-theory 3-form onto harmonic forms on $L_n$, to obtain massless *U*(1) gauge fields on $\mathcal {W}_{11-n}$ of various *p*-form degrees. Our goal is to generalise this picture, by expanding the M-theory 3-form onto *all* cohomology classes of $L_n$, both free and torsional.

On a factorized spacetime such as (28), it is natural to start from objects (differential forms, cohomology classes, differential cohomology classes) defined on the two factors, and combine them into objects on the total space. Let

29 $$\begin{aligned} p_\mathcal {W}: \mathcal {M}_{11} \rightarrow \mathcal {W}_{11-n} \ , \qquad p_L : \mathcal {M}_{11} \rightarrow L_n \end{aligned}$$

be the projection maps onto the two factors of $\mathcal {M}_{11}$. For notational simplicity, we henceforth omit the pullback maps $p_\mathcal {W}^*$ and $p_L^*$ from various factorized expressions. For example,

30a $$\begin{aligned}&\text {if }\lambda \in \Omega ^r(\mathcal {W}_{11-n})\text { and }\omega \in \Omega ^s(L_n),&\lambda \wedge \omega&\text { is shorthand for } p_\mathcal {W}^*(\lambda ) \wedge p_L^*(\omega ) \ , \end{aligned}$$

30b $$\begin{aligned}&\text {if }a \in H^r(\mathcal {W}_{11-n};{\mathbb {Z}})\text { and }b \in H^s(L_n;{\mathbb {Z}}),&a \smile b&\text { is shorthand for } p_\mathcal {W}^*(a) \smile p_L^*(b) \ , \end{aligned}$$

30c $$\begin{aligned}&\text {if }\breve{a} \in \breve{H}^r(\mathcal {W}_{11-n})\text { and }\breve{b} \in \breve{H}^s(L_n),&\breve{a} \star \breve{b}&\text { is shorthand for } p_\mathcal {W}^*(\breve{a}) \star p_L^*(\breve{b}) . \end{aligned}$$

We observe that the naturality of the products $\smile$ and $\star$, together with (17), implies

31 $$\begin{aligned} R(\breve{a} \star \breve{b}) = R(\breve{a}) \wedge R(\breve{b}) \ , \quad I(\breve{a} \star \breve{b}) = I(\breve{a}) \smile I(\breve{b}) \ , \quad \text { for }\breve{a} \in \breve{H}^r(\mathcal {W}_{11-n}), \breve{b} \in \breve{H}^s(L_n) . \end{aligned}$$

For each $p=0,\dots ,n, H^p(L_n; {\mathbb {Z}})$ is a finitely generated Abelian group. We take the generators of $H^p(L_n; {\mathbb {Z}})$ to be

32 $$\begin{aligned} \begin{aligned} \text {free generators of }H^p(L_n ;{\mathbb {Z}}):&\quad v_{p(\alpha )} , \quad \alpha \in \{ 1 , \dots b^p \} \\ \text {torsion generators of }H^p(L_n ;{\mathbb {Z}}):&\quad t_{p(i)},\quad i \in {\mathcal {I}}_p . \end{aligned} \end{aligned}$$

The subscript *p* is a reminder that these are classes of degree *p*, while $(\alpha ), (i)$ are labels that enumerate the generators. We define $b^p :=\dim H^p(L_n;{\mathbb {R}})$, the *p*-th Betti number of $L_n$. For the torsion generators, the index set $\mathcal {I}_p$ is some finite set of labels, which can be specified more explicitly in concrete examples. Each torsional generator has a definite torsional order: the minimal positive integer $n_{(i)}$ such that $n_{(i)} \, t_{p(i)} = 0$ (no sum on *i*).

For simplicity we take

33 $$\begin{aligned} \textrm{Tor} \, H^* (\mathcal {W}_{11-n} ; {\mathbb {Z}}) = 0 . \end{aligned}$$

By the Künneth formula, we may then expand a generic cohomology class $a_{4} \in H^4(\mathcal {M}_{11};{\mathbb {Z}})$ as

34 $$\begin{aligned} a_4 = \sum _{p=0}^4 \, \sum _{\alpha _p =1}^{b^p} \sigma _{4-p}^{(\alpha _p)} \smile v_{p(\alpha _p)} + \sum _{p=0}^4 \, \sum _{i_p \in \mathcal {I}_p } \rho _{4-p}^{(i_p)} \smile t_{p(i_p)} . \end{aligned}$$

In the above expression, $\sigma ^{(\alpha _p)}_{4-p}, \rho ^{(i_p)}_{4-p} \in H^{4-p}(\mathcal {W}_{11-d}; {\mathbb {Z}})$.

Recall that the map *I* in (8) is surjective. This applies both for elements in $\breve{H}^*(\mathcal {W}_{11-n})$ and $\breve{H}^*(L_n)$.^10^ It follows that there exist differential cohomology classes $\breve{F}_{4-p}^{(\alpha _p)}, \breve{B}_{4-p}^{(i_p)} \in \breve{H}_{4-p}(\mathcal {W}_{11-n})$ and $\breve{v}_{p(\alpha _p)}, \breve{t}_{p(i_p)} \in \breve{H}_p(L_n)$ such that

35 $$\begin{aligned} \sigma _{4-p}^{(\alpha _p)} = I ( \breve{F}_{4-p}^{(\alpha _p)} ) \ , \qquad \rho _{4-p}^{(i_p)} = I ( \breve{B}_{4-p}^{(\alpha _p)} ) \ , \qquad v_{p(\alpha _p)} = I (\breve{v}_{p(\alpha _p)}) \ , \qquad t_{p(i_p)} = I( \breve{t}_{p(i_p)} ) . \end{aligned}$$

With the objects $\breve{F}_{4-p}^{(\alpha _p)}, \breve{B}_{4-p}^{(i_p)} \in \breve{H}_{4-p}(\mathcal {W}_{11-n})$ and $\breve{v}_{p(\alpha _p)}, \breve{t}_{p(i_p)} \in \breve{H}_p(L_n)$ we can construct the following differential cohomology class

36 $$\begin{aligned} \breve{a}_4 = \sum _{p=0}^4 \, \sum _{\alpha _p =1}^{b^p} \breve{F}_{4-p}^{(\alpha _p)} \star \breve{v}_{p(\alpha _p)} + \sum _{p=0}^4 \, \sum _{i_p \in \mathcal {I}_p } \breve{B}_{4-p}^{(i_p)} \star \breve{t}_{p(i_p)} . \end{aligned}$$

The salient property of $\breve{a}_4$ in (36) is that it represents a possible lift of $a_4$ in (34), in the sense that

37 $$\begin{aligned} I(\breve{a}_4) = a_4 . \end{aligned}$$

This is verified using (35), the naturality of the differential cohomology product $\star$, and the second identity in (17).

The differential cohomology class $\breve{a}_4$ is not the most general class that reduces to $a_4$ under the action of *I*. From the discussion around (15), however, we know that any other class that reduces to $a_4$ must differ from $\breve{a}_4$ by a topologically trivial element of $\breve{H}^4(\mathcal {M}_{11})$, which can be represented by a globally defined 3-form. These considerations lead us to the following final form for the Ansatz for $\breve{G}_4$,

38 $$\begin{aligned} \breve{G}_4 = \sum _{p=0}^4 \, \sum _{\alpha _p =1}^{b^p} \breve{F}_{4-p}^{(\alpha _p)} \star \breve{v}_{p(\alpha _p)} + \sum _{p=0}^4 \, \sum _{i_p \in \mathcal {I}_p } \breve{B}_{4-p}^{(i_p)} \star \breve{t}_{p(i_p)} + \tau ([\omega _3]) \ , \qquad \omega _3 \in \Omega ^3(\mathcal {M}_{11}) . \end{aligned}$$

The first two sums in (38) encode all topological information about $\breve{G}_4$, while the last term collects the topologically trivial part of $\breve{G}_4$.

The differential cohomology classes $\breve{F}_{4-p}^{(\alpha _p)}, \breve{B}_{4-p}^{(i_p)} \in \breve{H}_{4-p}(\mathcal {W}_{11-n})$ encode external gauge fields. More precisely, we have:

- The class $\breve{F}_{4-p}^{(\alpha _p)}$, of degree $(4-p)$, represents a $(3-p)$-form gauge field with gauge group *U*(1), which restricts to a background field for a *U*(1) $(2-p)$-form symmetry on the boundary;
- The class $\breve{B}_{4-p}^{(i_p)}$, of degree $(4-p)$, represents a discrete $(4-p)$-form gauge field with gauge group $\mathbb Z_{n_{(i_p)}}$, where $n_{(i_p)}$ is the torsion order of $t_{p(i_p)}$, which restricts to a background field for a $\mathbb {Z}_{n_{(i_p)}}$ $(3-p)$-form symmetry on the boundary.

The first case is familiar, but the second one requires some additional explanation. Notice in particular the difference in the relation between the differential cohomology class degree and the degree of the higher form symmetry on the boundary. Consider two classes $\breve{B},\breve{B}'\in \breve{H}^{4-p}(\mathcal {W}_{11-d})$, such that $I(\breve{B})=I(\breve{B}')$. Then $I(\breve{B} - \breve{B}')=0$, so by exactness of (8) there is some globally defined differential form $\textsf{b}$ of degree $3-p$ such that $\breve{B}' = \breve{B} + \tau (\textsf{b})$. By (18) and naturality of $\tau$ and *R* we then have that $\tau (\textsf{b})\star \breve{t}_{p(i_p)}=\tau (\textsf{b}\wedge R(\breve{t}_{p(i_p)}))=0$, since we have chosen $\breve{t}$ to be flat. This implies $\breve{B}\star \breve{t}_{p(i_p)}=\breve{B}'\star \breve{t}_{p(i_p)}$, so $\breve{B}_{4-p}^{(i_p)}\star \breve{t}_{p(i_p)}$ is fully determined by its cohomology class $I(\breve{B}_{4-p}^{(i_p)})\smile I(\breve{t}_{p(i_p)})$ (given our canonical choice of $\breve{t}_{p(i_p)}$). This is an element of $H^{4-p}(\mathcal {W}_{11-d};\mathbb {Z})\otimes {{\,\textrm{Tor}\,}}H^{p}(L_p;\mathbb {Z})$, which by the universal coefficient theorem is isomorphic (since we are assuming ${{\,\textrm{Tor}\,}}H^{4-p}(\mathcal {W}_{11-d};\mathbb {Z})=0$) to $H^{4-p}(\mathcal {W}_{11-d}; {{\,\textrm{Tor}\,}}H^{p}(L_p;\mathbb {Z}))$. So by this isomorphism, we can reinterpret $I(\breve{B}_{4-p}^{(i_p)})\smile I(t_{p(i_p)})$ as a class in $H^{4-p}(\mathcal {W}_{11-d}; \mathbb {Z}_{n_{(i_p)}})$. But such a cohomology class is a map (up to homotopy) from $\mathcal {W}_{11-d}$ to the classifying space $K(\mathbb {Z}_{n_{(i_p)}}, 4-p)=B^{4-p}\mathbb {Z}_{n_{(i_p)}}$, which is the data that defines a principal bundle for a $(3-p)$-form symmetry. For instance, when $p=3$ we have an ordinary (0-form) discrete symmetry, and the backgrounds for such symmetries are maps from $\mathcal {W}_{11-d}$ to $B\mathbb {Z}_{n_{(i_p)}}$, or equivalently elements of $H^1(\mathcal {W}_{11-d};\mathbb {Z}_{n_{(i_p)}})$.

***Integration on products.***

Finally, we want to integrate differential cohomology classes on product spaces. Assume that $\breve{a}\in \breve{H}^p(X), \breve{b}\in \breve{H}^q(Y)$. Then

39 $$\begin{aligned} \int _{X\times Y} \breve{a} \star \breve{b} = (-1)^{(q-\dim (Y))\dim (X)}\left( \int _X\breve{a}\right) \star \left( \int _Y\breve{b}\right) . \end{aligned}$$

For the Chern–Simons coupling (26) we have $p+q=\dim (X)+\dim (Y)+1$. In this case:

40 $$\begin{aligned} \int _{X\times Y} \breve{a} \star \breve{b} = {\left\{ \begin{array}{ll} \left( \int _X u\right) \left( \int _Y R(\breve{b})\right) &{} \text {if } p=\dim (X)+1 ,\\ (-1)^p \left( \int _X R(\breve{a})\right) \left( \int _Y v\right) &{} \text {if } p=\dim (X) ,\\ 0 &{} \text {otherwise} , \end{array}\right. } \end{aligned}$$

simply by taking into account that the integrals on the right hand side of (39) are only non-vanishing for very specific values of *p* and *q*, as explained above. Here we have used that in the first case $\breve{a}$ is flat for degree reasons, so there is some $u\in H^{\dim (X)}(X; \mathbb {R}/\mathbb {Z})$ such that $\breve{a} = i(u)$, and similarly $\breve{b} = i(v)$ in the second case.

In this paper we are particularly interested in those integrals over the internal space involving torsional elements $\breve{t}$ (we omit the subindices here for notational simplicity). First note that since we have chosen these torsional generators to be flat, $R(\breve{t})=0$, we have $\tau ([\omega _3])\star \breve{t}=\tau ([\omega _3\wedge R(\breve{t})])=0$, due to (18). So any integral involving the $\breve{t}$ generators will be a topological invariant (including invariant under deformations of the connection), by virtue of being independent of $\tau ([\omega _3])$.

This implies that when expanding $\breve{G}_4^3$ using (38) we have

41 $$\begin{aligned} \breve{G}_4^3 = \sum \text {monomials involving }\breve{t}\text { and }\breve{v} + \sum \text {monomials involving }\breve{v}\text { and } \tau ([\omega _3]) , \end{aligned}$$

with no monomials involving both $\tau ([\omega _3])$ and the torsional classes. The second class of monomials are accessible using the ordinary formalism based on differential forms, so we will not discuss them further; both because they are well understood and because our interest in this paper is on discrete higher form symmetries, which arise from the torsional sector.

Now, regarding the first class of terms in (41), by (17) we have for all $\breve{a}=\breve{t}\star \breve{b}$ that $R(\breve{a})=0$, for any $\breve{b}$. (Note that by (17) $I(\breve{a})$ is automatically torsion if $I(\breve{t})$ is.) This implies that when doing the integration over the internal space $L_n$, torsional elements $\breve{a} = \breve{t}\star \breve{b}$ only contribute if $\breve{a}\in \breve{H}^{n+1}(L_n)$. By (40) this leads to effective actions on $\mathcal {W}_{11-d}$ which are primary invariants, not secondary ones. (Said more plainly: reducing the Chern–Simons term in 11d on the torsional sector leaves us with an ordinary integral of characteristic classes in $\mathcal {W}_{11-d}$.)

We now turn to deriving these theories, which will be the SymTFTs, in various geometric engineering settings.

## Symmetry TFTs from M-Theory on $S^3/\Gamma$

Consider first the case where the theory $\mathfrak {T}$ that we are engineering is a seven dimensional $\mathcal {N}=1$ theory with Lie algebra $\mathfrak {g}_\Gamma$, obtained by putting M-theory on $\mathcal {M}_{11}=\mathcal {M}_7\times \mathbb {C}^2/\Gamma$, where $\Gamma$ is a discrete subgroup of *SU*(2), and $\mathcal {M}_7$ is a (closed and torsion-free, for simplicity) manifold where $\mathfrak {T}$ lives. The resulting seven dimensional theory has defect group [59] $Z(G_\Gamma )^{(1)}\oplus Z(G_\Gamma )^{(4)}$ with $Z(G_\Gamma )$ the center of universal cover group $G_\Gamma$. For instance, the field theory with gauge group $G_\Gamma$ has electric 1-form symmetry $Z(G_\Gamma )$, while the theory with gauge group $G_\Gamma /Z(G_\Gamma )$ has magnetic 4-form symmetry $Z(G_\Gamma )$. Other global forms for the gauge group are often possible, depending on the choice of $\Gamma$, the analysis of the possibilities is identical to the one in [15]. The seven dimensional theory has additionally a 2-form *U*(1) instanton symmetry, with generator the integral of the instanton density on a closed 4-surface.

In the rest of this section we will argue that reducing M-theory on $S^3/\Gamma =\partial (\mathbb {C}^2/\Gamma )$ leads to an eight dimensional TFT encoding both this choice of global form for the seven dimensional theory (equivalently, the choice of its higher form symmetries) and the anomalies of these higher form symmetries.

We expect these two sectors of the eight dimensional TFT to interact in interesting ways: recall that choosing a global form for the gauge group (which will be able to rephrase as a choice of boundary behaviour in the BF theory (48)) can also be understood as a gauging of the higher form symmetries [16]. In the presence of ’t Hooft anomalies this gauging procedure might be obstructed, or lead to less conventional symmetry structures (see for instance [3, 7] for systematic discussions). It would be very interesting to analyse this problem from our higher dimensional vantage point, where it requires study of gapped boundary conditions of the TFTs we construct, but we will not do so in this paper.

### Choice of global structure from 8d

We start by discussing how to see the choice of global form in terms of a choice of boundary conditions for a gapped eight dimensional theory. The basic idea is discussed in [22–24], where it was found that the geometric origin of the 1-form and 4-form symmetries, and the fact that there is a choice to be made, can be traced back to the non-commutativity of the boundary values of $F_4$ and $F_7$ fluxes in the presence of torsion in the asymptotic boundary $\partial \mathcal {M}_{11}$ at infinity [27, 28].

Consider M-theory compactified on $\mathbb {R}\times X_{10}$, where we take the first factor to be the time direction. For the moment we ignore the Chern–Simons terms in the M-theory action, and view M-theory as a generalised Maxwell theory. We will discuss the effect of the Chern–Simons terms extensively below. We focus on the operators $\Phi (\mathcal {T}_3)$ and $\Phi (\mathcal {T}_6)$ measuring the periods of $C_3$ and $C_6$ on torsional cycles $\mathcal {T}_3$ and $\mathcal {T}_6$ inside $X_{10}$ (our discussion is only sensitive to the homology class of the cycles). The authors of [27, 28] found that these two operators do not always commute, but rather there is a phase in their commutation relation:

42 $$\begin{aligned} \Phi (\mathcal {T}_3)\Phi (\mathcal {T}_6) = e^{2\pi i\, \textsf{L}(\mathcal {T}_3,\mathcal {T}_6)} \Phi (\mathcal {T}_6)\Phi (\mathcal {T}_3) . \end{aligned}$$

Here, *L* is the linking pairing

43 $$\begin{aligned} \textsf{L}: {{\,\textrm{Tor}\,}}H_3(X_{10};\mathbb {Z}) \times {{\,\textrm{Tor}\,}}H_6(X_{10};\mathbb {Z}) \rightarrow \mathbb {Q}/\mathbb {Z}\, . \end{aligned}$$

This pairing can be defined as follows. Assume that $\mathcal {T}_6\in {{\,\textrm{Tor}\,}}H_6(X_{10};\mathbb {Z})$ is of order $n\in \mathbb {Z}$. That is, there is some chain $C_7$ such that $\partial C_7 = n \mathcal {T}_6$. Then

44 $$\begin{aligned} \textsf{L}(\mathcal {T}_3, \mathcal {T}_6) :=\frac{1}{n} \mathcal {T}_3 \cdot C_7 \mod 1 . \end{aligned}$$

One can analogously define a linking pairing ${{\,\textrm{Tor}\,}}H^k(\mathcal {M}_d;\mathbb {Z})\times {{\,\textrm{Tor}\,}}H^{d+1-k}(\mathcal {M}_d;\mathbb {Z})\rightarrow \mathbb {Q}/\mathbb {Z}$ in cohomology, which will appear below.

In our case we are putting M-theory on $\mathcal {M}_7\times \mathbb {C}^2/\Gamma$, which is not quite of the form $\mathbb {R}\times X_{10}$, but [22–24] argue that taking the radial direction of $\mathbb {C}^2/\Gamma$ as time (that is, taking $X_{10}=\mathcal {M}_7\times S^3/\Gamma$) reproduces the answers one expects from field theory. Our task is to modify the analysis in those papers to adapt it to the viewpoint that we are advocating here. In this section we will focus on the fate of the $\Phi (\mathcal {T}_3)$ and $\Phi (\mathcal {T}_6)$ operators upon dimensional reduction. The Chern–Simons part of the M-theory action gives additional contributions, which we study in detail below. We will find that reducing M-theory on $S^3/\Gamma$ leads to a non-invertible theory in eight dimensions whose states naturally correspond to the possible choices of global structure for the seven dimensional theory.

**Table 1:** The abelian group $H_1(S^3/\Gamma ;\mathbb {Z})=\Gamma ^{\text {ab}}$ and the linking pairing $\textsf{L}:\Gamma ^{\text {ab}}\times \Gamma ^{\text {ab}}\rightarrow \mathbb {Q}/\mathbb {Z}$, from [22]

$\Gamma$	$G_\Gamma$	$\Gamma ^{\text {ab}}$	$\textsf{L}_{S^3/\Gamma }$
$A_{n-1}$	*SU*(*n*)	$\mathbb {Z}_n$	$\frac{1}{n}$
$\textrm{D}_{2n}$	$\textrm{Spin}(4n)$	$\mathbb {Z}_2\oplus \mathbb {Z}_2$	$${1\over 2}\left( \begin{matrix} n &{} n-1 \\ n-1 &{} n \end{matrix}\right) $$
$\textrm{D}_{2n+1}$	$\textrm{Spin}(4n+2)$	$\mathbb {Z}_4$	$\frac{2n-1}{4}$
2*T*	$E_6$	$\mathbb {Z}_3$	$\frac{2}{3}$
2*O*	$E_7$	$\mathbb {Z}_2$	$\frac{1}{2}$
2*I*	$E_8$	0	0

We have altered the presentation of the results with respect to that paper to bring the table closer to its spin Chern–Simons refinement presented in Table 3 below

The analysis goes as follows. We place M-theory on a manifold of the form $\mathbb {R}\times \mathcal {M}_7\times S^3/\Gamma$, where we view the $\mathbb {R}$ factor as time when quantising. We are assuming that ${{\,\textrm{Tor}\,}}^k(\mathcal {M}_7)=0$ for all *k*, so by the Künneth formula

45 $$\begin{aligned} {{\,\textrm{Tor}\,}}H_p(X_{10};\mathbb {Z}) = H_{p-1}(\mathcal {M}_7;\mathbb {Z})\otimes H_1(S^3/\Gamma ;\mathbb {Z}) , \end{aligned}$$

where we are using the fact that the torsion in the homology of $S^3/\Gamma$ is localised on degree 1:

46 $$\begin{aligned} H_q(S^3/\Gamma ;\mathbb {Z}) = H^{3-q}(S^3/\Gamma ;\mathbb {Z}) = {\left\{ \begin{array}{ll} \mathbb {Z} &{} \text {for } q=0,3 ,\\ \Gamma ^{\text {ab}} :=\frac{\Gamma }{[\Gamma , \Gamma ]} &{} \text {for } q=1 ,\\ 0 &{} \text {for } q=2 . \end{array}\right. } \end{aligned}$$

The values for $\Gamma ^{\text {ab}}$ when $\Gamma \subset SU(2)$ are given in table 1. Looking to this table, one observes [22, 59, 74] that $\Gamma ^{\text {ab}}=Z(G_\Gamma )$, which is a key fact necessary for the whole picture to be consistent.

We find, in particular, that the torsional cycles $\mathcal {T}_3$ and $\mathcal {T}_6$ are necessarily of the form $\mathcal {T}_3=\Sigma _2\times \mathcal {T}_1$ and $\mathcal {T}_6=\Sigma _5\times \mathcal {T}_2$, with $\mathcal {T}_1$ and $\mathcal {T}_2$ generators of the torsional group $H_1(S^3/\Gamma ;\mathbb {Z})$, and $[\Sigma _p]\in H_p(\mathcal {M}_7;\mathbb {Z})$. Assume for simplicity that $\Gamma ^{\text {ab}}=\mathbb {Z}_k$, for some *k*. (The $D_{2n}$ cases can be treated similarly, at the cost of introducing more fields.) Then we have that from the point of view of the eight dimensional theory on $\mathbb {R}\times \mathcal {M}_7$ we have two kinds of operators $\Psi (\Sigma _2)$ and $\Psi (\Sigma _5)$, parametrised by two-surfaces $\Sigma _2$ and five-surfaces $\Sigma _5$. These operators are the eight dimensional push-forwards of the operators $\Phi (\Sigma _2\times \mathcal {T}_1)$ and $\Phi (\Sigma _2\times \mathcal {T}_1)$ in the eleven dimensional theory, with $\mathcal {T}_1$ the generator of $H_1(S^3/\Gamma ;\mathbb {Z})$. The commutation relation of these operators on a spatial slice $\mathcal {M}_7$ of the eight dimensional theory is determined from (42), using that $\textsf{L}(\Sigma _2\times \mathcal {T}_1, \Sigma _2\times \mathcal {T}_1) = (\Sigma _1\cdot \Sigma _2) \textsf{L}_{S^3/\Gamma }(\mathcal {T}_1, \mathcal {T}_1)$

47 $$\begin{aligned} \Psi (\Sigma _2)\Psi (\Sigma _5) = e^{2\pi i \ell ^{-1} \, \Sigma _2\cdot \Sigma _5} \Psi (\Sigma _5)\Psi (\Sigma _2) , \end{aligned}$$

where we have defined $\ell ^{-1}:=\textsf{L}_{S^3/\Gamma }(\mathcal {T}_1,\mathcal {T}_1)$.^11^ This is precisely the operator content and commutation relations of a BF theory with the following Lagrangian (see [25] for a derivation)

48 $$\begin{aligned} S_{\text {Sym}}= \ell \int B_2 \wedge dC_5 . \end{aligned}$$

For $|\ell | > 1$ this is a non-invertible theory, whose state space reproduces the choices of global structure expected from the field theory side [15, 22, 25, 75].

### Anomaly theory from link reduction

We will now describe how to obtain the eight dimensional anomaly theory encoding the mixed ’t Hooft anomaly between 1-form center symmetries and the 2-form *U*(1) instanton symmetry. We will find that the anomaly theory on any (closed and torsion-free, for simplicity) $\mathcal {W}_8$ can be derived by taking the eleven dimensional Chern–Simons part of the M-theory action on $\mathcal {W}_8\times S^3/\Gamma$, and integrating over $S^3/\Gamma$.

We are assuming ${{\,\textrm{Tor}\,}}(H^3(\mathcal {W}_8;\mathbb {Z}))=0$, so by the universal coefficient theorem $H^2(\mathcal {W}_8; \Gamma ^{\text {ab}})=H^2(\mathcal {W}_8;\mathbb {Z})\otimes H^2(S^3/\Gamma ;\mathbb {Z})$, and we can parametrize a generic element $a_4\in H^4(M^{11};\mathbb {Z})$ by

49 $$\begin{aligned} a_4 = \sigma _4\smile 1 + \sum _{i} \rho _2^{(i)} \smile t_{2(i)} + \sigma _1\smile \text {vol}(S^3/\Gamma ) . \end{aligned}$$

where $\text {vol}(S^3/\Gamma )$ is the generator of $H^3(S^3/\Gamma ;\mathbb {Z})=\mathbb {Z}, t_{2(i)}$ are the (torsional) generators of $H^{2}(S^3/\Gamma ;\mathbb {Z})=\Gamma ^{\text {ab}}$, and “1” is the generator of $H^0(S^3/\Gamma ;\mathbb {Z})=\mathbb {Z}$.

Given an element $a_4\in H^4(\mathcal {M}^{11};\mathbb {Z})$ written as (49) we can uplift to a differential cohomology class by using (38) to write

50 $$\begin{aligned} \breve{G}_4 = \breve{\gamma }_4 \star \breve{1} + \sum _i \breve{B}_2^{(i)}\star \breve{t}_{2 (i)} + \breve{\xi }_1\star \breve{v}_3 + \tau ([\omega _3]) , \end{aligned}$$

where $\omega _3\in \Omega ^3(\mathcal {M}^{11}), \breve{\xi }_1\in \breve{H}^1(\mathcal {W}_8;\mathbb {Z}), \breve{v}_3 \in \breve{H}^3(S^3/\Gamma ;\mathbb {Z})$, $I(\breve{\xi }_1)=\sigma _1$ and $I(\breve{v})=\text {vol}(S^3/\Gamma )$. We have $I(\breve{\gamma }_4)=\sigma _4$ and we choose $\breve{t}_{2(i)}$ such that $R(\breve{t}_{2(i)})=0$.

For the problem at hand, the terms in $\breve{I}_{12}$ in (27) originating from $X_8$ do not contribute for degree reasons. (They will play an important role in the case of 5d SCFTs below.) For simplicity of exposition, let us assume first that $\Gamma ^{\textrm{ab}}$ has a single generator. Substituting (50) into the Chern–Simons coupling (26) we obtain

51 $$\begin{aligned} \begin{aligned} \frac{S_{\text {top}}}{2\pi }&=-\frac{1}{6} \int _{M_{11}} \breve{G}_4 \star \breve{G}_4 \star \breve{G}_4 \\&= \frac{1}{2} \int _{\mathcal {W}_8} \breve{\gamma }_4^2 \star \breve{\xi }_1 \int _{S^3/\Gamma } \breve{v}- \frac{1}{2} \int _{\mathcal {W}_8} \breve{\gamma }_4 \star \breve{B}_2^2 \int _{S^3/\Gamma } \breve{t}_2^2- \frac{1}{6} \int \breve{\tau }(w_3) . \end{aligned} \end{aligned}$$

The first term in this expression encodes a potential mixed anomaly between a $(-1)$-form symmetry and the 2-form instanton symmetry. The second term in (51) corresponds to a mixed ’t Hooft anomaly between the center 1-form symmetry $Z(G_\Gamma )=\Gamma ^{\text {ab}}$ and the instanton 2-form symmetry. In what follows we will concentrate on this last term. To find the coefficient of this anomaly, we must evaluate the integral

52 $$\begin{aligned} \textrm{CS}[S^3/\Gamma , \breve{t}_2] = \frac{1}{2} \int _{S^3/\Gamma } \breve{t}_2\star \breve{t}_2 . \end{aligned}$$

This ${\mathbb {R}}/{\mathbb {Z}}$-valued quantity is the spin Chern–Simons invariant, evaluated for the 3-manifold $S^3/\Gamma$ and the flat connection $\breve{t}_2 \in \breve{H}^2(S^3/\Gamma ;\mathbb {Z})$. In general such Chern–Simons invariants also depend on the spin structure on the manifold. In our case, by construction, we have the spin connection induced on the boundary of the supersymmetric compactification of M-theory on $\mathbb {C}^2/\Gamma$.

Using $\int _{S^3/\Gamma } \breve{v} = 1$, and neglecting the $\tau$ term according to the general discussion of Sect. 2.3, we compute the SymTFT:

53 $$\begin{aligned} \boxed {S_{\text {Sym}}= \frac{1}{2} \, \int _{\mathcal {W}_8} \breve{\gamma }_4 \star \breve{\gamma }_4 \star \breve{\xi }_1 - \textrm{CS}[S^3/\Gamma , \breve{t}_2] \, \int _{\mathcal {W}_8} \breve{\gamma }_4 \star \breve{B}_2 \star \breve{B}_2 .} \end{aligned}$$

The cases in which $\Gamma ^{\textrm{ab}}$ has two generators can be treated in a completely analogous way, yielding

54 $$\begin{aligned} S_{\text {Sym}}= \frac{1}{2} \, \int _{\mathcal {W}_8} \breve{\gamma }_4 \star \breve{\gamma }_4 \star \breve{\xi }_1 - \sum _{i,j}\textrm{CS}[S^3/\Gamma ]_{ij} \, \int _{\mathcal {W}_8} \breve{\gamma }_4 \star \breve{B}_2^{(i)} \star \breve{B}_2^{(j)} . \end{aligned}$$

Note that formally one would be tempted to write

55 $$\begin{aligned} \textrm{CS}[S^3/\Gamma ]_{ij} = \frac{1}{2}\int _{S^3/\Gamma } \breve{t}_{2(i)} \star \breve{t}_{2(j)} . \end{aligned}$$

The factor of $\frac{1}{2}$ makes the right hand side not well defined. Luckily (but unsurprisingly, given that our starting Chern–Simons coupling in M-theory action is well-defined [69]), due to the symmetry properties (17) of the Cheeger–Simons product is only the sum $\textrm{CS}[S^3/\Gamma ]_{ij}+\textrm{CS}[S^3/\Gamma ]_{ji}$ that enters in the anomaly theory (54), and this sum is well defined:

56 $$\begin{aligned} \textrm{CS}[S^3/\Gamma ]_{ij} + \textrm{CS}[S^3/\Gamma ]_{ji} = \int _{S^3/\Gamma } \breve{t}_{2(i)} \star \breve{t}_{2(j)} . \end{aligned}$$

Similar remarks apply to the off-diagonal entries in the $D_{2n}$ case in table 3 below. We provide a more systematic discussion of this issue at the end of this section.

### Evaluation of the Chern–Simons invariant

Let us now discuss a convenient formalism to evaluate the CS invariant (52) (including the 1/2 prefactor), obtained by a straightforward generalisation of a discussion in the three dimensional case by Gordon and Litherland [76].

#### Cohomology of the link and bulk compact divisors

Let $L_{d-1}$ be a closed, connected, oriented $(d-1)$-manifold, and suppose that $L_{d-1}$ can be realised as boundary of a *d*-manifold $X_{d}$. The long exact sequence in relative homology yields

57 $$\begin{aligned} \dots \rightarrow H_{d-2}(X_d;\mathbb {Z})&\rightarrow H_{d-2}(X_d,L_{d-1};\mathbb {Z}) \rightarrow H_{d-3}(L_{d-1};\mathbb {Z})\nonumber \\&\rightarrow H_{d-3}(X_d;\mathbb {Z}) \rightarrow \dots . \end{aligned}$$

We now make the assumption

58 $$\begin{aligned} H_{d-3}(X_d;\mathbb {Z}) = 0 . \end{aligned}$$

Using Poincaré duality in $L_{d-1}$ we have $H_{d-3}(L_{d-1};\mathbb {Z}) \cong H^2(L_{d-2};\mathbb {Z})$, and from (57) we get the exact sequence

59 $$\begin{aligned} H_{d-2}(X_d;\mathbb {Z}) \xrightarrow {A} H_{d-2}(X_d, L_{d-1};\mathbb {Z}) \xrightarrow {f} H^2(L_{d-1};\mathbb {Z}) \rightarrow 0 . \end{aligned}$$

Notice in particular that the homomorphism *f* is surjective: any class in $H^2(L_{d-1};\mathbb {Z})$ can be lifted to an element in $H_{d-2}(X_d, L_{d-1};\mathbb {Z})$. Let us now consider a torsional class $a_2 \in H^2(L_{d-1};\mathbb {Z})$, satisfying $n a_2 = 0$ for some positive integer *n*. We know that there exists an element $\kappa \in H_{d-2}(X_d, L_{d-1};\mathbb {Z})$ such that $f(\kappa ) = a_2$. Since *f* is a homomorphism, $0 = n a_2 = nf(\kappa ) = f(n \kappa )$, i.e. $n \kappa \in \ker f$. Exactness of the sequence (59) implies that there exists an element $Z\in H_{d-2}(X_d;\mathbb {Z})$ such that $A (Z) = n \kappa$.

**Fig. 2:**
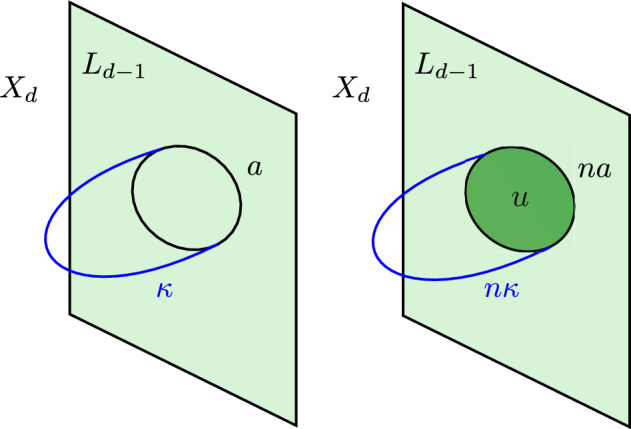
On the left: Under the assumption $H_{d-3}(X_d;\mathbb {Z}) =0$, any $(d-3)$-cycle $a \in Z_{d-3}(L_{d-1})$ in the link can be realised as boundary of a $(d-2)$-chain $\kappa \in C_{d-2}(X_d)$ in the bulk $X_d$. On the right: If *a* represents a torsional homology class, $na = \partial u$ for some $(d-2)$-chain $u\in C_{d-2}(L_{d-1})$ in the link, which can also be naturally regarded as an element in $C_{d-2}(X_d)$. Combining the chains *u* and $n\kappa$ we get a cycle, $\partial (n\kappa - u) = 0$. This cycle can now be smoothly retracted to the interior of $X_d$, and can therefore be thought of as a compact cycle. Its homology class $[n\kappa -u]$ represents $Z \in H_{d-2}(X_d;\mathbb {Z})$

The manifolds $L_{d-1}$ that we want to study are the link of a canonical Calabi–Yau singularity $X^{\text {singular}}_d$, so in our case there is a very natural family of choices of $X_d$: we can take any crepant resolution of $X^{\text {singular}}_d$. Relatedly, we refer to elements of $H_p(X_d, L_{d-1};\mathbb {Z})$ as non-compact *p*-cycles in $X_d$, and to elements of $H_p(X_d;\mathbb {Z})$ as compact *p*-cycles in $X_d$. The observations made so far can be summarised as follows:

- To every class $a_2 \in H^2(L_{d-1};\mathbb {Z})$ we can associate a non-compact $(d-2)$-cycle $\kappa$ in $X_d$.
- To every torsional class $a_2 \in H^2(L_{d-1};\mathbb {Z})$ we can associate a compact $(d-2)$-cycle *Z* in $X_d$ via the following relations,
  60 $$\begin{aligned} n a_2 = 0 \ , \qquad a_2 = f(\kappa ) \ , \qquad A (Z) = n \kappa \ , \end{aligned}$$
  where $\kappa$ is a non-compact $(d-2)$-cycle in $X_d$ (Fig. 2).

Let us now analyse the map *A* in (59) in greater detail. By Lefschetz duality,

61 $$\begin{aligned} H_{d-2}(X_d, L_{d-1};\mathbb {Z}) \cong H^2(X_d;\mathbb {Z}) . \end{aligned}$$

To proceed, we make the further assumption (that holds in all the cases in this paper)

62 $$\begin{aligned} {\textrm{Tor}} \, H_1(X_d;\mathbb {Z}) = 0 . \end{aligned}$$

The universal coefficient theorem then guarantees that

63 $$\begin{aligned} H^2(X_d;\mathbb {Z}) \cong \textrm{Hom} (H_{2}(X_d;\mathbb {Z}) , {\mathbb {Z}}) . \end{aligned}$$

We may then recast (59) in the form

64 $$\begin{aligned} H_{d-2}(X_d;\mathbb {Z}) \xrightarrow {A} \textrm{Hom} (H_{2}(X_d;\mathbb {Z}) , \mathbb Z) \xrightarrow {f} H^2(L_{d-1};\mathbb {Z}) \rightarrow 0 . \end{aligned}$$

The homomorphism *A* can be equivalently regarded as a bilinear $\mathbb Z$-valued pairing between $H_{2}(X_d;\mathbb {Z})$ and $H_{d-2}(X_d;\mathbb {Z})$,

65 $$\begin{aligned} A : H_{d-2}(X_d;\mathbb {Z}) \otimes H_2(X_d;\mathbb {Z}) \rightarrow {\mathbb {Z}} . \end{aligned}$$

Indeed, *A* is identified with the intersection pairing of compact $(d-2)$-cycles and compact 2-cycles in $X_d$. Once we choose a basis for $H_{2}(X_d;\mathbb {Z})$ and $H_{d-2}(X_d;\mathbb {Z})$, the map *A* is represented by the intersection matrix $\mathcal {M}_{d-2,2}$.

#### Evaluation for $S^3/\Gamma$

To compute the SymTFT for the 7d theory (53) we need to evaluate the CS invariant. Let us now specialise to a 3d link $L_3$, and fix a class $t_2 \in \textrm{Tor} \,H^2(L_3;\mathbb {Z})$ such that $n t_2 =0$. Let $Z \in H_2(X_4;\mathbb {Z})$ be the compact 2-cycle in $X_4$ associated to $t_2$. By the discussion above, the linking pairing of (the Poincaré dual to) $t_2$ with itself can be computed as

66 $$\begin{aligned} \int _{L_3} \breve{t}_2 \star \breve{t}_2 = {\textsf{L}}_{L_3} ( \textrm{PD}[t_2] ,{\textrm{PD}}[t_2]) = \bigg [ \frac{Z \cdot Z}{n^2} \bigg ]_\text {mod 1} . \end{aligned}$$

In the above expression, $\cdot$ denotes the intersection pairing among compact 2-cycles in $X_4$. Our task is actually to compute a CS invariant of the form (52). In the Gordon-Litherland approach, this quantity is given by

67 $$\begin{aligned} {\textrm{CS}}[L_3 , \breve{t}_2] = \frac{1}{2} \int _{L_3} \breve{t}_2\star \breve{t}_2 = \bigg [ \frac{Z \cdot Z}{2\,n^2} \bigg ]_\text {mod 1} \, . \end{aligned}$$

**Table 2:** The center divisors *Z* written in terms of the compact curves associated to the simple roots for ADE-singularities

$\Gamma$	Dynkin diagram	*Z*
$A_{n-1}$		$\sum _{i=1}^{n-1} i S_i$
$\textrm{D}_{2n}$		$\sum _{i=1}^{2n-1}(1-(-1)^i)S_i$
$\textrm{D}_{2n+1}$		$$\begin{matrix} {1\over 2} \sum _{i=1}^{2n-1}(1-(-1)^i) S_i ,\\ {1\over 2} \sum _{i=1}^{2n-2}\left( {1-(-1)^i}\right) S_i+S_{2n} \end{matrix}$$
$E_6$		$\sum _{i=1}^5 iS_i+S_{2n}+3S_{2n+1}$
$E_7$		$S_1+S_3+S_7$

We omit the $E_8$ case as it has trivial center symmetry

In particular, we apply this formalism to the case of interest $L_3 = S^3/\Gamma$. The bulk $X_4$ can be chosen to be the resolved ALE space ${\mathbb {C}}^2/\Gamma$. We notice that the assumptions (58) and (62) are indeed satisfied. The intersection matrix $\mathcal {M}_{2,2}$ representing the map *A* equals minus the Cartan matrix of the Lie algebra ${\mathfrak {g}}_\Gamma$. For the ADE-singularities, the choice of central divisors which gives the center of the gauge group has been identified in [37]. We list their results in table 2. Using these compact divisors as representatives of the torsional generators in order to be able to compare easily with known field theory results,^12^ we obtain the results given in table 3 for the spin Chern–Simons invariant $\textrm{CS}[S^3/\Gamma , \breve{t}_2]$. It is a nice check of our formalism that the resulting coefficients in the anomaly theory perfectly reproduce the answer one gets from a purely field theory analysis [48]. (The $A_{n-1}$ answer was also recently obtained in [57] from a related viewpoint.)

**Table 3:** $\Gamma$ of ADE type. $G_\Gamma$ denotes the simply-connected gauge group in 7d, $\Gamma ^{ab}$ the abelianization of $\Gamma$

$\Gamma$	$G_\Gamma$	$\Gamma ^{\text {ab}}$	$-\textrm{CS}[S^3/\Gamma , \breve{t}_2]$
$A_{n-1}$	*SU*(*n*)	$\mathbb {Z}_n$	$\frac{n-1}{2n}$
$\textrm{D}_{2n}$	$\textrm{Spin}(4n)$	$\mathbb {Z}_2\oplus \mathbb {Z}_2$	$${1\over 4}\left( \begin{matrix} n &{} n-1 \\ n-1 &{} n \end{matrix}\right) $$
$\textrm{D}_{2n+1}$	$\textrm{Spin}(4n+2)$	$\mathbb {Z}_4$	$\frac{2n+1}{8}$
2*T*	$E_6$	$\mathbb {Z}_3$	$\frac{5}{3}$
2*O*	$E_7$	$\mathbb {Z}_2$	$\frac{3}{4}$
2*I*	$E_8$	0	0

Finally $\textrm{CS}[S^3/\Gamma , \breve{t}_2]$ is the Chern–Simons invariant (52), which is closely related to the linking pairing, as explained in the main text. With these expressions one can then evaluate the SymTFT for 7d SYM in (53)

***Relation to the Linking Pairing.***

Finally, we want to comment on the relation between the Chern–Simons invariant $\text {CS}[S^3/\Gamma , \breve{t}_2]$ and the linking pairing $\textsf{L}(t_2, t_2)$. The relation is that $\text {CS}[S^3/\Gamma , \breve{t}_2]$ provides a quadratic refinement [65, 69] of the linking pairing:

68 $$\begin{aligned} \textsf{L}_{S^3/\Gamma } ( \textrm{PD}[s_2],\textrm{PD}[t_2])= & {} \int _{S^3/\Gamma } \breve{s}_2 \star \breve{t}_2 = \text {CS}[S^3/\Gamma , \breve{s}_2+\breve{t}_2] - \text {CS}[S^3/\Gamma , \breve{s}_2] \nonumber \\{} & {} - \text {CS}[S^3/\Gamma , \breve{t}_2] \mod 1, \end{aligned}$$

where $\breve{s}_2,\breve{t}_2\in \breve{H}^2(S^3/\Gamma )$ are chosen to be flat: $R(\breve{s}_2)=R(\breve{t}_2)=0$. The equality on the left can be proven as follows.

Since $\breve{s}_2$ and $\breve{t}_2$ are flat, we can use the commutativity of (8), and (18) to write

69 $$\begin{aligned} \breve{s}_2\star \breve{t}_2 = -\breve{s}_2 \star i(\beta ^{-1}(I(\breve{t}_2))) = - i\left( I(\breve{s}_2) \smile \beta ^{-1}(I(\breve{t}_2))\right) , \end{aligned}$$

where we have also used that $H^1(S^3/\Gamma ; \mathbb {R})=H^2(S^3/\Gamma ;\mathbb {R})=0$, so the Bockstein map $\beta$ in (8) is an isomorphism. For the integral of (69) we then have

70 $$\begin{aligned} \int _{S^3/\Gamma } i\left( I(\breve{s}_2) \smile \beta ^{-1}(I(\breve{t}_2))\right)= & {} i\left( \int _{S^3/\Gamma } I(\breve{s}_2) \smile \beta ^{-1}(I(\breve{t}_2))\right) \nonumber \\= & {} \int _{S^3/\Gamma } I(\breve{s}_2) \smile \beta ^{-1}(I(\breve{t}_2)) , \end{aligned}$$

since $i: H^0(\text {pt}; U(1)) \rightarrow \breve{H}^1(\text {pt})$ is an isomorphism. The final expression in (70) is just the linking pairing $\textsf{L}$ on $S^3/\Gamma$ [77] (up to a sign convention)

71 $$\begin{aligned} \int _{S^3/\Gamma } \breve{s}_2\star \breve{t}_2 = -\int _{S^3/\Gamma } I(\breve{s}_2) \smile \beta ^{-1}(I(\breve{t}_2)) = \textsf{L}_{S^3/\Gamma }(\textrm{PD}[s_2] , \textrm{PD}[t_2]) \mod 1 . \end{aligned}$$

This refinement of the linking pairing extends, in particular, the observation in [22] that the fractional instanton number for an instanton bundle in the presence of background 1-form flux is half of the linking pairing in $S^3/\Gamma$ for the torsional class $t_2$ representing the 1-form flux background. The more refined statement that follows from our M-theory construction is instead:

72 $$\begin{aligned} n_{\text {inst}} = -\text {CS}[S^3/\Gamma , \breve{t}_2] \mod 1 . \end{aligned}$$

The discussion in [22] was specific to four dimensional theories on Spin manifolds, and the two statements agree on that class of manifolds (up to an overall sign that was chosen oppositely in [22]), but (72) gives the correct answer on non-Spin manifolds too.

## Symmetry TFTs for 5d SCFTs

Placing M-theory on toric Calabi–Yau threefold singularities leads to a rich and interesting class of five dimensional SCFTs. This is a very active area of investigation, started by [78–82]. These theories have an intricate set of global symmetries, starting with 0-form flavor symmetries, which are enhanced at the UV fixed point, as well as discrete higher-form symmetries—both 1-form (or 2-form) symmetries [23, 24, 37] and 3-form symmetries [54, 55]. These symmetries can have ’t Hooft anomalies, and by gauging some of the symmetries one obtains theories with 2-group structure [3, 83].

In this section we will determine a subset of the six dimensional symmetry TFT by reducing M-theory on the five-dimensional Sasaki–Einstein manifold $L_5$ linking the singular point. In Sect. 5 below we will present an alternative derivation based on reducing the Chern–Simons terms on the worldvolume of the dual system on (*p*, *q*) 5-branes down to six dimensions. One important omission from our analysis is the sector leading to 2-group structures, which we leave for future work.

### 5d SCFTs from M-theory and higher form symmetries

We will start by giving a summary of the salient features of 5d SCFT engineering from M-theory that will play a role in this paper. We are interested in the dynamics of M-theory on a singular toric Calabi-Yau- threefold *X*. The global 0-form flavor symmetries are encoded in the non-compact divisors $\{D_i\}$ of *X* and their intersections with the compact divisors $S_a, a= 1, \cdots , r$, which furnish the Cartan generators of the gauge group on the Coulomb branch (CB), of rank *r*. The general flavor symmetry on the CB contains an instanton $U(1)_I$, whose current is $j_I= * {{\,\textrm{Tr}\,}}(F\wedge F)$, and which often in the UV enhances the flavor symmetry compared to the gauge theory description in the IR. Many geometric methods of computing the UV flavor symmetry from the non-compact divisors have been developed [84–91], which enable computing also the global form of the flavor symmetry groups [83].

The higher form symmetries arise from the homology groups of $L_5:=\partial X$. The 2-form symmetry $\Gamma ^{(2)}$ under which the ’t Hooft surface operators are charged, is determined by the group [22–24]

73 $$\begin{aligned} \Gamma ^{(2)} = {H_2(X, L_5; \mathbb {Z} ) \over H_2 (X; \mathbb {Z})} \cong H_1 (L_5; \mathbb {Z}) , \end{aligned}$$

where the last isomorphism is true if all 1-cycles in $L_5$ trivialise in the bulk (this will be true for *X* simply because $H_1(X;\mathbb {Z})=0$). In the cases of interest to us this group will be of the form $\mathbb {Z}_k$ for some *k* that depends on *X*.

As noted in [22–24], this group can be computed in any smooth resolution of *X* using the intersection of compact divisors and compact curves in the Calabi–Yau by

74 $$\begin{aligned} \Gamma ^{(1)} \cong \mathbb {Z}^{b_4}/\mathcal {M}_{4,2} \mathbb {Z}^{b_2} , \end{aligned}$$

where the Betti numbers are related to the rank of the CB gauge group *r* and flavor rank *f* by $b_2 =r+f$ and $b_4=r$. $\mathcal {M}_{4,2} = (S_i \cdot _X C_k)$ is the intersection matrix of compact divisors $S_i$ with compact curves $C_k$. Alternatively, it can be computed without needing to resolve *X* by looking to the structure of the external points in the toric diagram [24, 92].

Similarly, as explained in Sect. 2.3, the torsional part of $H_3 (L_5;\mathbb {Z})$ will lead to a finite abelian 1-form symmetry under which line operators are charged, while the free projection of $H_3 (L_5;\mathbb {Z})$ leads to a continuous 0-form symmetry, which includes the instanton symmetry or $U(1)_I$.

These higher form symmetries are not all realised simultaneously in a given field theory. As in the seven dimensional case studied above, we will obtain a generically non-invertible BF sector in the symmetry theory when reducing on $L_5$, and different choices of boundary conditions for the symmetry theory will determine which higher form symmetries are actually realised. The derivation of the BF sector is very similar to the one in that case, so we will be brief. Consider the operators $\Phi (\mathcal {T}_3)$ and $\Phi (\mathcal {T}_6)$ wrapped on generators $t_1$ and $t_3$ of $H_1(L_5;\mathbb {Z})$ and ${{\,\textrm{Tor}\,}}H_3(L_5;\mathbb {Z})$. They will lead to operators $\Psi (\Sigma _2)$ and $\Psi (\Sigma _3)$ in the effective six dimensional symmetry theory, with a commutation relation

75 $$\begin{aligned} \Psi (\Sigma _2)\Psi (\Sigma _3) = e^{2\pi i \ell ^{-1} \Sigma _2\cdot \Sigma _3} \Psi (\Sigma _3)\Psi (\Sigma _2) \end{aligned}$$

on a spatial slice, where $\ell ^{-1}:=\textsf{L}_{L_5}(t_1, t_3)$. This is the content of a BF theory with action^13^

76 $$\begin{aligned} S_{\text {BF}} = \ell \int B_2\wedge dC_3 . \end{aligned}$$

The 1-form symmetries also participate in ’t Hooft anomalies. Denote the background fields for the 1-form symmetry by $$B_2 \in H^2 (M_5; \Gamma ^{(1)})$$. From general field theory considerations, obtained by studying the Coulomb branch, there are two types of anomalies: the purely 1-form symmetry cubic anomaly ($$B^3 $$) [58], and the mixed $$U(1)_I$$ and 1-form symmetry anomaly ($$B^2 F_I$$) [63]. The cubic 1-form symmetry anomaly was derived from field theory in the context of the SCFTs that have a Coulomb branch description as $$SU(p)_q$$ [58]

77 $$\begin{aligned} \mathcal {A}_{B^3} = {q p (p-1) (p-2) \over 6 \gcd (p,q)^3} B_2^3 . \end{aligned}$$

We are using conventions where the periods of $B_2$ are integrally quantised (as opposed to $2\pi /\gcd (p,q)$ quantised) and the 1-form symmetry in this case is $\Gamma ^{(1)} = \mathbb {Z}_{\gcd (p,q)}$. This coupling corresponds to a ’t Hooft anomaly for the 1-form symmetry, and therefore field theoretically obstructs its gauging. This will imply that (potentially) some asymptotic flux choice might be obstructed, and not all the global form of the gauge group are allowed, unless a more complicated structure arises, which mixes the 1-form symmetry with other symmetries present in the theory. We plan to explore deeper consequences of this coupling by using our methods in the future.

There is also a field theoretic mixed anomaly between the instanton $U(1)_I$ and 1-form symmetry, determined in [63] for the $SU(2)_0$ theory using field theory arguments. In the IR for $SU(p)_q$ it takes the form [58, 63]

78 $$\begin{aligned} \mathcal {A}_{FB^2} = {p (p-1)\over 2 \gcd (p,q)^2} F_I B_2^2 . \end{aligned}$$

(This contribution to the anomaly was also analysed in [57] using string theory methods, reaching a different conclusion. We believe that the discrepancy between their result and ours might be due to a different choice of torsional representative, see footnote 12 above.)

We will now derive these anomalies from first principles using the differential cohomology approach developed in this paper, being agnostic about whether this is a UV or IR computation. We will see that an essential contribution for these anomalies comes from the $C_3 \wedge X_8$ term in the M-theory effective action.

### Link reduction using differential cohomology

The integral cohomology of $L_5$, the base of the toric Calabi–Yau cone *X*, takes the form

79 $$\begin{aligned} H^*(L_5;{\mathbb {Z}}) = \big \{ {\mathbb {Z}} , 0 , {\mathbb {Z}}^{b^2} \oplus \textrm{Tor}\, H^2(L_5;{\mathbb {Z}}) , {\mathbb {Z}}^{b^2} \oplus \textrm{Tor}\, H^3(L_5;{\mathbb {Z}}) , \textrm{Tor}\, H^2(L_5;{\mathbb {Z}}) , {\mathbb {Z}} \big \} . \end{aligned}$$

For simplicity, we assume that the Betti number $b^1$ of $L_5$ is zero. (This is true in all examples we study.) The expansion of $\breve{G}_4$ then reads

80 $$\begin{aligned} \breve{G}_4&= \breve{\gamma }_4 \star \breve{1} + \sum _{\alpha =1}^{b^2} \breve{F}_2^{(\alpha )} \star \breve{v}_{2(\alpha )} + \sum _{\alpha = 1}^{b^2} \breve{\xi }_{1(\alpha )} \star \breve{v}_3 ^{(\alpha )} \nonumber \\&\quad + \sum _i \breve{B}_2^{(i)} \star \breve{t}_{2(i)} + \sum _m \breve{b}_1^{(m)} \star \breve{t}_{3(m)} + \sum _i \breve{\psi }_{0(i)} \star \breve{t}_4^{(i)} + \tau ([\omega _3]) . \end{aligned}$$

The label $\alpha$ runs over generators of the free part of $H^2(L_5;{\mathbb {Z}})$, the label *i* runs over generators of $\textrm{Tor} \, H^2(L_5;{\mathbb {Z}}_5)$, while the label *m* runs over generators of $\textrm{Tor} \, H^3(L_5;{\mathbb {Z}})$.

We can now consider the reduction of the $G_4^3$ coupling in M-theory. Using (80) and collecting all relevant terms, we arrive at

81 $$\begin{aligned} \begin{aligned}&- \frac{1}{6} \, \int _{\mathcal {M}_{11}} \breve{G}_4 \star \breve{G}_4 \star \breve{G}_4 \\&\quad = - \sum _\alpha \int _{\mathcal {W}_6} \breve{\gamma }_4 \star \breve{F}_2^{(\alpha )} \star \xi _{1(\alpha )} - \sum _{i,j,k}\bigg [ \frac{1}{6} \, \int _{L_5} \breve{t}_{2(i)} \star \breve{t}_{2(j)} \star \breve{t}_{2(k)} \bigg ] \\&\qquad \int _{\mathcal {W}_6} \breve{B}_2^{(i)} \star \breve{B}_2^{(j)} \star \breve{B}_2^{(k)} \\&\quad - \sum _{i,j,\alpha } \bigg [\frac{1}{2} \, \int _{L_5} \breve{t}_{2(i)} \star \breve{t}_{2(j)} \star \breve{v}_{2(\alpha )} \bigg ] \, \int _{\mathcal {W}_6} \breve{B}_2^{(i)} \star \breve{B}_2^{(j)} \star \breve{F}_2^{(\alpha )}\\&\quad - \sum _{i,\alpha ,\beta } \bigg [ \frac{1}{2} \, \int _{L_5} \breve{t}_{2(i)} \star \breve{v}_{2(\alpha )} \star \breve{v}_{2(\beta )} \bigg ] \, \int _{\mathcal {W}_6} \breve{B}_2^{(i)} \star \breve{F}_2^{(\alpha )} \star \breve{F}_2^{(\beta )} \\&\quad + \sum _{m,n}\bigg [ \frac{1}{2} \, \int _{L_5} \breve{t}_{3(m)} \star \breve{t}_{3(n)} \bigg ] \, \int _{\mathcal {W}_6} \breve{\gamma }_4 \star \breve{b}_1^{(m)} \star \breve{b}_1^{(n)} \\&\quad + \sum _{m,\alpha } \bigg [ \int _{L_5} \breve{t}_{3(m)} \star \breve{v}_3^{(\alpha )} \bigg ] \, \int _{\mathcal {W}_6} \breve{\gamma }_4 \star \breve{b}_1^{(m)} \star \breve{\xi }_{1(\alpha )} \\&\quad - \sum _{i,j} \bigg [ \int _{L_5} \breve{t}_{2(i)} \star \breve{t}_4^{(j)} \bigg ] \, \int _{\mathcal {W}_6} \breve{\gamma }_4 \star \breve{B}_2^{(i)} \star \breve{\psi }_{0(j)} \\&\quad - \sum _{\alpha ,j}\bigg [ \int _{L_5} \breve{v}_{2(\alpha )} \star \breve{t}_4^{(j)} \bigg ] \, \int _{\mathcal {W}_6} \breve{\gamma }_4 \star \breve{F}_2^{(\alpha )} \star \breve{\psi }_{0(j)} . \end{aligned} \end{aligned}$$

In the first term, we have used $\int _{L_5} v_{2(\alpha )} \star v_3^{(\beta )} = \delta ^\beta _\alpha$.

Next, let us consider the terms that originate from the $G_4 X_8$ coupling in M-theory. Recall that 11d spacetime is taken to be the direct product $\mathcal {M}_{11} = \mathcal {W}_6 \times L_5$. As a result, at the level of cohomology classes with integer coefficients, one has^14^

84 $$\begin{aligned} \begin{aligned} p_1(T\mathcal {M}_{11})&= p_1(T\mathcal {W}_6) + p_1(TL_5) \\ p_2(T\mathcal {M}_{11})&= p_2(T\mathcal {W}_6) + p_2(TL_5) + p_1(T\mathcal {W}_6) \smile p_1(TL_5) . \end{aligned} \end{aligned}$$

These relations imply

85 $$\begin{aligned} X_8 = - \frac{1}{96} \, p_1(T\mathcal {W}_6) \smile p_1(TL_5) . \end{aligned}$$

Promoting integral cohomology classes to differential cohomology classes (the precise representative of $p_1$ one chooses is not important [94]) we can write the $G_4 X_8$ coupling in the form

86 $$\begin{aligned} - \int _{\mathcal {M}_{11}} \breve{G}_4 \star \breve{X}_8= & {} \frac{1}{96} \, \int _{\mathcal {M}_{11}} \breve{G}_4 \star \breve{p}_1(T\mathcal {W}_6) \star \breve{p}_1(TL_5)\nonumber \\= & {} \frac{1}{96} \, \sum _i \int _{L_5} \breve{t}_{2(i)} \star \breve{p}_1(TL_5) \, \int _{\mathcal {W}_6} \breve{B}_2^{(i)} \star \breve{p}_1(T\mathcal {W}_6) . \end{aligned}$$

In the second step we used (80) and we observed that the only internal differential cohomology classes that can have a non-trivial pairing with $\breve{p}_1(TL_5)$ are the degree-2 torsional classes $\breve{t}_{2(i)}$.

To proceed, we make use of the following congruence for integral cohomology classes [95]

87 $$\begin{aligned} p_1(T\mathcal {W}_6) \smile a_2 = 4 \, a_2 \smile a_2 \smile a_2 \mod 24 \qquad \text {for any }a_2 \in H^2(\mathcal {W}_6 ; {\mathbb {Z}}) . \end{aligned}$$

This congruence can be derived using the Atiyah-Singer index theorem as follows [96]. We take external spacetime $\mathcal {W}_6$ to be a Spin manifold. Consider an arbitrary $a_2 \in H^2(\mathcal {W}_6; {\mathbb {Z}})$. There exists a line bundle with connection *A* on $\mathcal {W}_6$ such that its first Chern class equals $a_2$. Consider the Dirac operator on $\mathcal {W}_6$ twisted by this line bundle. The Atiyah-Singer theorem implies88where *F* is the curvature 2-form of the connection *A*, satisfying $[F]_{\textrm{dR}} = \varrho (a_2)$. We conclude that $\int _{\mathcal {W}_6} [ 4\,a_2 \smile a_2 \smile a_2 - a_2 \smile p_1(T\mathcal {W}_6) ] \in 24\,{\mathbb {Z}}$, which is equivalent to (86).

Relation (86) then implies

82 $$\begin{aligned} \begin{aligned} \int _{\mathcal {W}_6} \breve{B}_2^{(i)} \star \breve{p}_1(T\mathcal {W}_6)&= \int _{\mathcal {W}_6} B_2^{(i)} \smile p_1(T\mathcal {W}_6) = 24\, M^{(i)} + 4\, \int _{\mathcal {W}_6} B_2^{(i)} \smile B_2^{(i)} \smile B_2^{(i)} \\&= 24 \, M^{(i)} + 4 \, \int _{\mathcal {W}_6} \breve{B}_2^{(i)} \star \breve{B}_2^{(i)} \star \breve{B}_2^{(i)} \ , \end{aligned}\end{aligned}$$

where we have used the fact that $\int _{\mathcal {W}_6} \breve{a}_6 = \int _{\mathcal {W}_6} I(\breve{a}_6)$ for any $\breve{a}_6 \in \breve{H}^6(\mathcal {W}_6)$, together with (17), $I(\breve{B}_2^{(i)}) = B_2^{(i)}$, and $I( \breve{p}_1(T\mathcal {W}_6)) = \breve{p}_1(T\mathcal {W}_6)$. The quantities $M^{(i)}$ are unspecified integers, encoding the ambiguity in the mod 24 congruence (86). There is no summation on the repeated label *i* in (88). Inserting (88) into (85), we arrive at

89 $$\begin{aligned}{} & {} - \int _{\mathcal {M}_{11}} \breve{G}_4 \star \breve{X}_8 = \frac{1}{24} \, \sum _i \int _{L_5} \breve{t}_{2(i)} \star \breve{p}_1(TL_5) \, \int _{\mathcal {W}_6} \breve{B}_2^{(i)} \star \breve{B}_2^{(i)} \star \breve{B}_2^{(i)}\nonumber \\{} & {} \quad + \sum _i M^{(i)} \, \int _{L_5} \breve{t}_{2(i)} \star \frac{\breve{p}_1(TL_5)}{4} . \end{aligned}$$

In Appendix A we show that $p_1(TL_5)/4$ is an integral class. Although we have no general proof, we also find that in all of our examples the quantity multiplying $M^{(i)}$ is an integer, so we will drop the last term in what follows, and focus on the terms in the symmetry theory that contain only the fields $\breve{B}_2^{(i)}$ and $\breve{F}_2^{(\alpha )}$.

Notice that there is an ambiguity in the definition of the differential cohomology classes $\breve{v}_{2(\alpha )}$ associated to the free part of $H^2(L_5;{\mathbb {Z}})$, which can be shifted by integral multiples of the differential cohomology classes $\breve{t}_{2(i)}$ associated to $\textrm{Tor}\,H^2(L_5;{\mathbb {Z}})$, $\breve{v}_{2(\alpha )} \rightarrow \breve{v}_{2(\alpha )} + m_{(\alpha )}{}^{(i)} \, \breve{t}_{2(i)}$, with $m_{(\alpha )}{}^{(i)} \in {\mathbb {Z}}$. Our choices are such that for the examples in the paper we have

90 $$\begin{aligned} \int _{L_5} \breve{t}_{2(i)} \star \breve{v}_{2(\alpha )} \star \breve{v}_{2(\beta )} = 0 . \end{aligned}$$

Combining (81) and (89), we obtain the following anomaly couplings in the symmetry TFT:

91 $$\begin{aligned} \boxed { S_{\text {Sym}}= \sum \limits _{i,j,k} \Omega _{ijk} \, \int _{\mathcal {W}_6} \breve{B}_2^{(i)} \star \breve{B}_2^{(j)} \star \breve{B}_2^{(k)} + \sum \limits _{i,j,\alpha } \Omega _{ij\alpha } \, \int _{\mathcal {W}_6} \breve{B}_2^{(i)} \star \breve{B}_2^{(j)} \star \breve{F}_2^{(\alpha )} ,} \end{aligned}$$

where the ${\mathbb {R}}/{\mathbb {Z}}$-valued quantities $\Omega _{ijk}$, $\Omega _{ij\alpha }$ are CS invariants defined by

92 $$\begin{aligned} \begin{aligned} \Omega _{ijk}&=4 \frac{1}{6} \, \int _{L_5} \breve{t}_{2(i)} \star \breve{t}_{2(j)} \star \breve{t}_{2(k)} + \frac{1}{24} \, \delta _{i,j} \, \delta _{i,k} \, \int _{L_5} \breve{t}_{2(i)} \star \breve{p}_1(TL_5) \\ \Omega _{ij\alpha }&= - \frac{1}{2} \, \int _{L_5}\breve{t}_{2(i)} \star \breve{t}_{2(j)} \star \breve{v}_{2(\alpha )} . \end{aligned} \end{aligned}$$

As we demonstrate in Appendix A, for the setups of interest in this work $G_4$ is integrally quantised, and therefore the 11d couplings in the M-theory effective action are guaranteed to be well-defined. It follows that the CS invariants (92) are also well-defined. Let us emphasize, however, that the two terms in $\Omega _{ijk}$ with $i=j=k$ are not separately well-defined, in general.

The CS invariants $\Omega _{ijk}, \Omega _{ij\alpha }$ are defined purely in terms of the link geometry $L_5$. In order to evaluate them for a given $L_5$, however, it can be convenient to resort to a computation in the bulk of the Calabi–Yau $X_6$, using an extension of the Gordon-Litherland formalism discussed in Sect. 3.3. Let $n_{(i)}$ denote the torsional degree of $t_{2(i)} \in H^2(L_5;\mathbb {Z})$, and let $Z_{(i)}$ be the compact divisor in the bulk associated to $t_{2(i)}$. We also associate a non-compact divisor $D_{(\alpha )}$ to the non-torsional classes $v_{2(\alpha )} \in H^2(L_5;\mathbb {Z})$, which correspond to flavor symmetries. With this notation, the invariants (92) can be computed as

93 $$\begin{aligned} \begin{aligned} \Omega _{ijk}&= \bigg [ - \frac{1}{6} \, \frac{Z_{(i)} \cdot Z_{(j)} \cdot Z_{(k)} }{n_{(i)} \, n_{(j)} \, n_{(k)} } + \frac{1}{24} \, \delta _{i,j} \, \delta _{i,k} \, \frac{Z_{(i)} \cdot p_1(TX_6) }{n_{(i)}} \bigg ]_\text {mod 1} \ , \\ \Omega _{ij\alpha }&= \bigg [ - \frac{1}{2} \, \frac{Z_{(i)} \cdot Z_{(j)} \cdot D_{(\alpha )} }{ n_{(i)} \, n_{(j)} } \bigg ]_\text {mod 1} \ , \end{aligned} \end{aligned}$$

where $\cdot$ denotes intersection of divisors in $X_6$.

### Examples: $SU(p)_q$ from $Y^{p,q}$

**Fig. 3:**
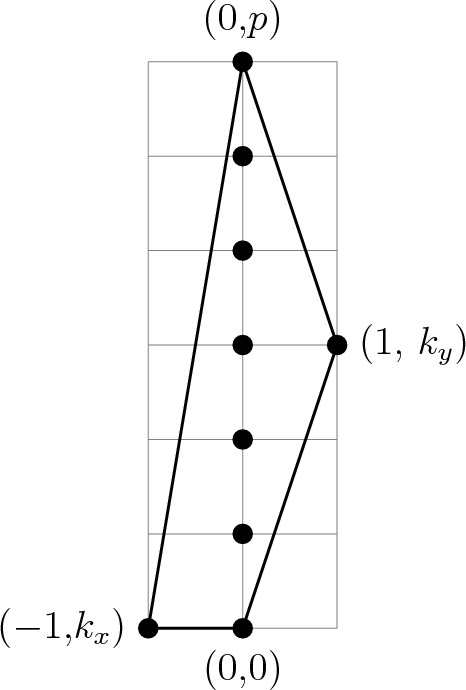
The toric diagram for the 5d SCFT realization of $SU(p)_q$. The example shown is $p=6, q= 6-(k_x + k_y) =3$, i.e. $SU(6)_3$, which has $\mathbb {Z}_3$ 1-form symmetry

This general approach can be exemplified for all toric Calabi–Yau cones, in particular the SCFTs with $SU(p)_q$ IR description, which have from field theory analysis, the anomalies in (77) and (78). The Sasaki–Einstein link is given by $Y^{p,q}$, and the Calabi–Yau has simple toric description: the toric diagram for $SU(p)_q$ is given by Fig. 3. For a detailed discussion of this geometry see e.g. [97]. The external vertices that determine the toric fan are

94 $$\begin{aligned} w_0= (0,0), \qquad w_p = (0,p) ,\qquad w_{x} = (-1, k_x) ,\qquad w_y = (1, k_y) , \end{aligned}$$

where the CS-level *q* is determined by

95 $$\begin{aligned} q= p-(k_x + k_y) . \end{aligned}$$

There are linear relations among these non-compact divisors $D_{w_i}$, and the instanton *U*(1) is identified with

96 $$\begin{aligned} D_{I} = D_{w_x} . \end{aligned}$$

The compact divisors are

97 $$\begin{aligned} S_a = (0,a) ,\qquad a= 1, \cdots , p-1 . \end{aligned}$$

As shown in [37] the center symmetry generator of the gauge theory *SU*(*p*) is obtained by taking the linear combination

98 $$\begin{aligned} Z = \sum _{a=1}^{p-1} a S_a . \end{aligned}$$

This compact divisor is also identified with the compact divisor associated to the generator of $\textrm{Tor} \, H^2(L_5;\mathbb {Z})$ according to the discussion in Sect. 3.3.

We will also need an explicit expression for $p_1(TX_6)=-c_2(TX_6\otimes \mathbb {C})$, with $TX_6\otimes \mathbb {C}$ the complexification of the tangent bundle of the toric Calabi–Yau $X_6$. Since $TX_6$ is a complex vector bundle we have $TX_6\otimes \mathbb {C}=TX_6\oplus \overline{TX_6}$, so $c(TX_6\otimes \mathbb {C})=c(TX_6)c(\overline{TX_6})$. For a toric variety $X_6$ with divisors $D_i$ we have [98]

99 $$\begin{aligned} c(TX_6) = \prod _{i=1}^n (1+D_i) \end{aligned}$$

so

100 $$\begin{aligned} c(TX_6\otimes \mathbb {C}) = c(TX_6)c(\overline{TX_6}) = \left( \prod _{i=1}^n (1+D_i)\right) \left( \prod _{i=1}^n (1-D_i)\right) = \prod _{i=1}^n (1-D_i^2) . \end{aligned}$$

and therefore $$p_1(TX_6)=\sum _i D_i^2$$.

With this information at hand it is straightforward to compute the anomaly coefficients using the formulae (93), specialised to the case of one torsion generator of order $\textrm{gcd}(p,q)$ and one free generator. We have done these computations with Sage [99] for $p<20$ and $|q|\le p$, and find results compatible with the empirical formulas

101 $$\begin{aligned} \begin{aligned} Z \cdot Z \cdot Z&= p \, (p-1) \, (p^2 + p\,q - 2 \,q) \ , \qquad Z \cdot p_1 = 4 \, p \, (p-1) \ , \\ Z \cdot Z \cdot D_I&= -p \, (p-1) \ , \end{aligned} \end{aligned}$$

which we conjecture hold in general. We have also verified in a large class of examples that we always have $Z \cdot D_I \cdot D_I = 0$, in accordance to the general claim (90), and that

102 $$\begin{aligned} \int _{L_5} \breve{t}_2 \star \frac{\breve{p}_1(TL_5)}{4} = \bigg [ \frac{Z \cdot p_1}{4 \, \textrm{gcd}(p,q)} \bigg ]_\text {mod 1} =0 . \end{aligned}$$

This condition guarantees that the terms in (89) not fixed by the mod 24 congruence (86) can indeed be safely dropped.

Assuming the validity of (101) it is straightforward to verify that

103 $$\begin{aligned}{} & {} - \frac{1}{6} \, Z \cdot Z \cdot Z + \frac{1}{24} \, \textrm{gcd}(p,q) \, Z \cdot p_1 = \frac{q \, p \, (p-1) \, (p-2)}{6} \nonumber \\{} & {} \quad - \textrm{gcd}(p,q)^3 (p-1) \, \frac{P \, (P+1) \, (P-1)}{6} \, \end{aligned}$$

where $P = p/\textrm{gcd}(p,q)$. Plugging (101) in (93), and using (103), we find that the action for the symmetry TFT contains the terms

104 $$\begin{aligned} S_{\text {Sym}}= \int _{\mathcal {W}_6} \bigg [ \frac{q \, p \, (p-1) \, (p-2)}{6 \, \textrm{gcd}(p,q)^3} \, B_2^3 + \frac{p \, (p-1)}{2\, \textrm{gcd}(p,q)^2} \, B_2^2 \, F_I \bigg ] . \end{aligned}$$

This result is in perfect agreement with the field theory results (77) and (78). It may be worth noting that the result is well-defined, because it is invariant under shifts of $B_2$ by $\textrm{gcd}(p,q)$ times an arbitrary integral class. For example, if we perform the shift $B_2 \rightarrow B_2 + \textrm{gcd}(p,q) \, b_2$, the extra terms generated by the $B_2^3$ term are

105 $$\begin{aligned} \begin{aligned}&\frac{q \, p \, (p-1) \, (p-2)}{6 \, \textrm{gcd}(p,q)^3} \, 3 \, \textrm{gcd}(p,q) \, \int _{\mathcal {W}_6}B_2^2 \, b_2 \in {\mathbb {Z}} \ , \\ \\&\frac{q \, p \, (p-1) \, (p-2)}{6 \, \textrm{gcd}(p,q)^3} \, 3 \, \textrm{gcd}(p,q)^2 \, \int _{\mathcal {W}_6}B_2 \, b_2^2 \in {\mathbb {Z}} \ , \\\\&\frac{q \, p \, (p-1) \, (p-2)}{6 \, \textrm{gcd}(p,q)^3} \, \textrm{gcd}(p,q)^3 \, \int _{\mathcal {W}_6} b_2^3 \in {\mathbb {Z}} . \end{aligned} \end{aligned}$$

Similar remarks apply to the $B_2^2 \, F_I$ term.

### Examples: non-Lagrangian toric models

**Fig. 4:**
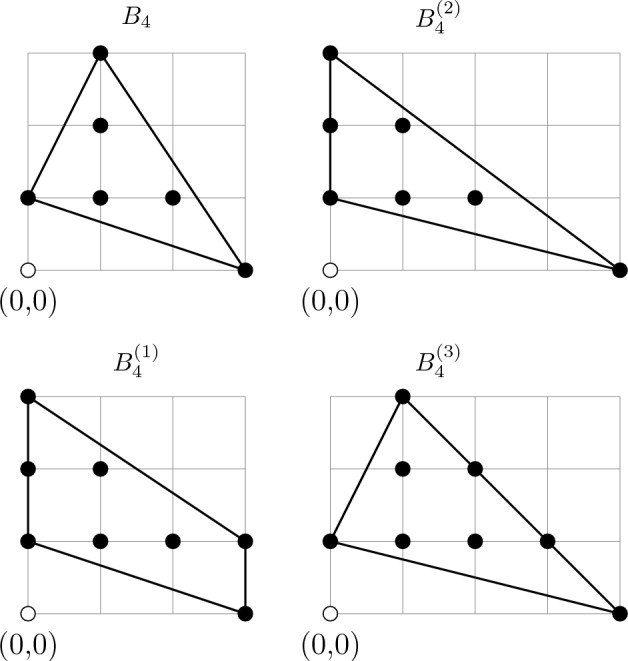
$B_N$ and $B_N^{(i)}$ non-Lagrangian toric diagrams for $N=4$

Our geometric approach becomes particularly useful when the theories in question do not have any non-abelian gauge theory description—i.e. are truly non-Lagrangian. As an illustration, we consider the toric models $B_N$ and $B_N^{(i)}$ introduced and studied in [23, 100], which do not have any non-abelian gauge theory description in 5d on the Coulomb branch. They are defined in terms of their toric fan in table 4, where also their 1-form symmetry is tabulated. Examples of the $N=4$ models are shown in Fig. 4. Note that $B_{N=3}$ is the rank 1 $\mathbb {P}^2$ Seiberg theory, which we show to also have a non-trivial 1-form symmetry anomaly.

**Table 4:** Properties of the $B_N$ and $B_N^{(i)}$ non-Lagrangian toric models

Theory	$\Gamma ^{(1)}$	Toric Fan
$B_N$	$\mathbb {Z}_{N (N-3)+ 3}$	$(N-1, 0,1), (1, N-1, 1), (0,1,1)$
$B_N^{(1)}$	$\mathbb {Z}_{N-1}$	$((N-1,0, 1), (N-1, 1, 1) (0, N-1-k, 1)), \, k= 0, \cdots , N-2$
$B_N^{(2)}$	$\mathbb {Z}_{N}$	$( (N,0, 1), (0, N-1-k, 1)), \, k= 0, \cdots , N-2$
$B_N^{(3)}$	$\mathbb {Z}_{N-1}$	$( (0,1, 1), (N-k, k, 1)), \, k= 0, \cdots , N-1$

We can again compute the $B^3$ terms in the SymTFT for $B_N$:

106 $$\begin{aligned} S_{\text {Sym}}^{(B_N)} = \int _{\mathcal {W}_6} {(N-1)(N-2) \over 6 (N(N-3)+3) } \, B_2^3 \end{aligned}$$

For $B_N^{(1)}$ we find

107 $$\begin{aligned} S_{\text {Sym}}^{(B_N^{(1)})} =\int _{\mathcal {W}_6} \frac{(N-3) (N-2)}{6 (N-1)} \, B_2^3 . \end{aligned}$$

For $B_N^{(2)}$ we find

108 $$\begin{aligned} S_{\text {Sym}}^{(B_N^{(2)})} =\int _{\mathcal {W}_6} \frac{(N-2) (N-1)}{6 N} \, B_2^3 . \end{aligned}$$

Finally for $B_N^{(3)}$ the $B_2^3$ anomaly vanishes.

In computing these, we have picked a particular central divisor associated to the generator of the 1-form symmetry. e.g. in the case of $B_N^{(2)}$ theories, we conjecture this to be of the form

109 $$\begin{aligned} Z_{B^{(2)}_N} = (-N+1) \sum _{i=1}^{N-2} v_{1,i} + (-N+2) \sum _{i=1}^{N-3} v_{2,i} + \cdots . \end{aligned}$$

Since there is no non-abelian gauge theory description any central divisor is in fact equally acceptable, but this choice leads to simple general formulas.

Finally, let us note that the $B_N^{(1)}$ theories have a *U*(1) factor in their flavor symmetry groups [100]. We can compute the mixed anomaly between this *U*(1) symmetry and the 1-form symmetry. To this end, we use the general formula (93), specialised to the case of a single *Z* central divisor. The non-compact divisor *D* associated to the *U*(1) flavor symmetry is identified with the divisor associated to the vertex with coordinates $(N-1,0)$ in the toric diagram for $B_N^{(1)}$, see Fig. 4. We find the following additional term in the symmetry TFT:

110 $$\begin{aligned} S_{\text {Sym}}^{(B_N^{(1)}),\text {mixed}} = \int _{\mathcal {W}_6} \frac{N-2}{2 (N-1)} \, B_2^2 \, F , \end{aligned}$$

where *F* is the field strength of the background field for the *U*(1) flavor symmetry.

The application of our approach to this class of non-Lagrangian theories in 5d, demonstrates the flexibility and generality of the approach using our differential cohomology extension of dimensional reductions on the link. Here we focused on toric models, but any non-compact Calabi–Yau three-fold geometry that has a canonical singularity, i.e. 5d SCFT, can be studied in this way.

## 5d Anomalies from the Boundary of (*p*, *q*) 5-Brane Webs

We now consider IIB (*p*, *q*) 5-brane webs engineering 5d SCFTs, and in particular we would like to evaluate the IIB supergravity action at the boundary of these webs to compute a 6d bulk action. We focus on the part of this action which involves the 1-forms symmetries and corresponds to the anomalies of the 5d SCFTs.

### Mixed anomalies

In order to describe the boundary geometry, we assume that the topology of the near-horizon limit of the IIB (*p*, *q*) 5-brane webs at large *N* in [34] extends generically to all webs. This implies that the boundary is given by $M_4$ that is an $S^2$ fibered over a disc, $\Sigma$, with punctures at the boundary of $\Sigma$ representing the (*p*, *q*) 5-brane sources. The fibered $S^2$ shrinks at the boundary of the disc away from the punctures, and the full space is topologically equivalent to an $S^4$ with punctures. The non-trivial topological cycles are 3-cycles $\{c_{\ell }\}$, and the 3-form representatives of the third cohomology of the $M_4$ are denoted by $\{\beta _{\ell } = \nu _{\ell } \wedge \textrm{vol}(S^2)\}$, where $\ell = 1, \ldots , L$ is the total number of semi-infinite 5-brane stacks. $\nu _{\ell }$ corresponds to the angular direction around the punctures and $\beta _{\ell }$ the volume forms for of these 3-cycles. In particular $\{c_{\ell }\}$ need to satisfy the following linear relation [101]

111 $$\begin{aligned} \sum _{\ell } c_{\ell } = 0, \qquad \sum _{\ell } p_{\ell } c_{\ell }=0, \qquad \sum _{\ell } q_{\ell } c_{\ell }=0, \end{aligned}$$

which are dual to the relations among divisors in eq. (3.24) of [97] of the toric Calabi–Yau in the M-theory construction.

The are also non-trivial 1-cycles with volume forms given by $\{\omega _{\ell } \}$. Locally they can be thought as the hodge dual of the $\beta _{\ell }$, and they have to satisfy the same constraints (111).^15^ The intersection pairing reads

112 $$\begin{aligned} \int _{M_4} \omega _{\ell } \wedge \beta _{j} = \Omega _{\ell j} \text {Vol}_4 . \end{aligned}$$

We expand $$F_3, H_3, C_4$$

113 $$\begin{aligned} \begin{aligned}&F_3= q^{\ell } \beta _{\ell } + f_2^{\ell } \wedge \omega _{\ell } + g_3 + \ldots \\&H_3=p^{\ell } \beta _{\ell } + h_2^{\ell } \wedge \omega _{\ell }+ h_3 +\ldots \\&F_5= f_5+ f_4^{\ell } \wedge \omega _{\ell } + g_2^{\ell } \wedge \beta _{\ell } +f_1 \wedge \textrm{vol}_4 , \end{aligned} \end{aligned}$$

where self-duality of $F_5$ impose that $*_6 f_5 = f_1$ and $*_6 f_4 = f_2$. In addition there is a field strength for the pair $H_3,F_3$ expanded on $\omega _{\ell }$ corresponding to

114 $$\begin{aligned} da_{1}^{\ell } = \sum _j \Omega _{\ell j} (q^{\ell } h_2^{j} - p^{\ell } f_2^j). \end{aligned}$$

Not all of $a_{1}^{\ell }$ will be linearly independent, since the 1-cycles as well the 3-cycles, $c_{\ell }$, are also not all independent (111). The most relevant aspect is the counting of the massless vector fields which corresponds to the backgrounds of the abelian flavor symmetries, whereas the details of the intersection pairing $\Omega$, as long as it is non-trivial, will not affect the results in any significant way.

We study now the reduction of the IIB topological coupling, which we extend to a 11-dimensional coupling to ensure gauge invariance

115 $$\begin{aligned} S_{11}^{\textrm{top}} = \int F_5\wedge H_3 \wedge F_3 . \end{aligned}$$

The Bianchi identity $dF_5=H_3\wedge F_3$ imposes the following constraints in BF frame $(f_5,f_4)$,

116 $$\begin{aligned} \begin{aligned} df_5 = h_3 \wedge f_3&\ \rightarrow \ f_5 = dc_4+ b_2 \wedge f_3 \\ df_4^{\ell } = (h_3 \wedge f_2^{\ell } - g_3 \wedge h_2^{\ell })&\ \rightarrow \ f_4^{\ell } = dc_3^{\ell } + (b_2 f_2^{\ell } - c_2 h_2^{\ell }) \end{aligned} \end{aligned}$$

and in the Stückelberg frame $(f_1,f_2)$

117 $$\begin{aligned} \begin{aligned} df_1 = g_2^{\ell } q_{\ell } - g_2^{\ell } p_{\ell }&\ \rightarrow \ f_1= dc_0+ b_1^{\ell } q_{\ell } - c_1^{\ell } p_{\ell } \\ dg_2^{\ell } = (q^{\ell } h_3 - p^{\ell } g_3)&\ \rightarrow \ g_2^{\ell } = d{c}_1^{\ell } + (q^{\ell } b_2 - p^{\ell } c_2) . \end{aligned} \end{aligned}$$

***BF frame.***

From the expansion (113) and (116), we obtain various contributions to couplings. The first interesting coupling is given by the singleton theory, which can be recast in a $SL(2\mathbb {R})$ covariant way as follows,

118 $$\begin{aligned} S^{\textrm{singl}}_{7d} = \int dc_3^{j} \Omega _{j \ell } Q^{\ell I}\wedge \sigma _{IJ}\mathcal {F}^J_3, \end{aligned}$$

where

119 $$\begin{aligned} Q^{\ell I} = \begin{pmatrix} p_1 &{} q_1 \\ \vdots &{} \vdots \\ p_L &{} q_L \\ \end{pmatrix}, \qquad \mathcal {F}^J_3 = \begin{pmatrix} h_3 \\ g_3\end{pmatrix}, \qquad \sigma _{IJ}= \begin{pmatrix} 0 &{} 1\\ -1 &{} 0 \end{pmatrix}, \end{aligned}$$

by suppressing the indices, using matrix multiplication, and defining $C_3=\{c_3^1, \ldots , c_3^L \}$, we can write (118) as,

120 $$\begin{aligned} S^{\textrm{singl}}_{7d} = \int dC_3 \Omega Q \sigma \mathcal {F} = \int dC_3 \Omega B^{-1} B Q A A^{-1} \sigma \mathcal {F} = \int d \widetilde{C}_3 Q_{\textrm{SNF}} \widetilde{\mathcal {F}}_3, \end{aligned}$$

where $Q_{\textrm{SNF}} = B Q A$ is the Smith normal form of *Q* and *A*, *B* is the pair of matrices transforming *Q*, moreover we have that $d\widetilde{C}_3 = (B^{-1})^T \Omega dC_3$ and $\widetilde{\mathcal {F}}_3 = A^{-1} \sigma \mathcal {F}_3$. The study of boundary conditions and mutual locality of this action determines the 1 or 2-form symmetries of the theory.

We now study what happens to the vector fields $a_1^{\ell }$ with coupling

121 $$\begin{aligned} S_{7d}^{\textrm{vec}}=\int dc_4 \wedge (q^{\ell } \Omega _{\ell j} h_2^{j} - p^{\ell } \Omega _{\ell j} f_2^{j}). \end{aligned}$$

This can be written in an $SL(2,\mathbb {R})$ covariant way as follows

122 $$\begin{aligned} S_{7d}^{\textrm{vec}}= \int dc_4 \wedge \Omega _{j \ell } Q^{\ell I} \sigma _{IJ} \mathcal {F}_2^{I j} = \int dc_4 \wedge \text {Tr}(Q^T\Omega \mathcal {F}_2^T \sigma ) . \end{aligned}$$

where *Q* and $\sigma$ have been defined in (118)

123 $$\begin{aligned} \mathcal {F}_2 = \begin{pmatrix} h_2^1 &{} \ldots &{}h_2^L \\ f_2^1 &{} \ldots &{}f_2^L \end{pmatrix} . \end{aligned}$$

In particular (122) can be rewritten as follows,

124 $$\begin{aligned} \begin{aligned} S_{7d}^{\textrm{vec}}&=\int dc_4 \wedge \text {Tr}( (B^{-1} B Q A A^{-1})^T \Omega \mathcal {F}_2^T \sigma ) = \int dc_4 \wedge \text {Tr}((A^{-1})^T Q_{\textrm{SNF}}^T \widetilde{\mathcal {F}}_2^T \sigma )=\\&= \int dc_4 \wedge \text {Tr}(\sigma \widetilde{\mathcal {F}}_2 Q_{\textrm{SNF}} A^{-1} ), \end{aligned} \end{aligned}$$

where $\widetilde{\mathcal {F}}_2= (B^{-1})^T \Omega \mathcal {F}_2^T$. This is the dual of the Stückelberg mechanism, which makes a combination of the vectors massive, and the massless one satisfies,

125 $$\begin{aligned} \text {Tr}(\sigma \widetilde{\mathcal {F}}_2 Q_{\textrm{SNF}} A^{-1} )=0 . \end{aligned}$$

The number of linear independent vectors that are dual to *U*(1) flavor symmetries is 0 if $L=3$ and $2(L-3)-1$ otherwise.

Finally, we also have additional couplings, which could potentially lead to anomalies between the *U*(1) flavor symmetries and the 1-form symmetries, which in $SL(2,\mathbb {Z})$ coviariant form reads

126 $$\begin{aligned} S^{\textrm{anom}}_{7d}= & {} \int (q^{\ell } b_2 - p^{\ell } c_2) \wedge \Omega _{\ell j} (f_2^{j} h_3 - h_2^j f_3) = \int \mathcal {C}_2 \sigma Q^T \Omega \mathcal {F}_2^T \sigma \widetilde{\mathcal {F}}_3\nonumber \\= & {} \int \widetilde{\mathcal {C}}_2 Q_{\textrm{SNF}}^T \widetilde{\mathcal {F}}_2^T A \widetilde{\mathcal {F}}_3, \end{aligned}$$

where $$\mathcal {F}_3 = d\mathcal {C}_2$$ and $$\widetilde{\mathcal {F}}_3 = d\widetilde{\mathcal {C}}_2$$.

***Stückelberg frame.***

The same physical consequences hold also in Stückelberg frame. The second Bianchi identity in (117) implies that

127 $$\begin{aligned} g_2^{\ell }=d{\tilde{c}}_1^{\ell } + Q \sigma \mathcal {C}_2= d{\tilde{c}}_1^{\ell } + B^{-1} Q_{\textrm{SNF}} \widetilde{\mathcal {C}}_2 . \end{aligned}$$

The kinetic therm for $g_2^{\ell }$ could make some of the component $\widetilde{\mathcal {C}}_2$ discrete depending on the entries of $Q_{\textrm{SNF}}$. The first identity in (117) can be rewritten such that

128 $$\begin{aligned} f_1 = dc_0 + \text {Tr}( \sigma \mathcal {F}_2 \Omega Q ) = dc_0 + \text {Tr}(\sigma \widetilde{\mathcal {F}}_2 Q_{\textrm{SNF}} A^{-1} ) , \end{aligned}$$

leading to the same constraint given by (124). Moreover the expansion of (115), leads to the same expression (126) for the mixed anomaly between the 1-form symmetry and the *U*(1) flavors. We will see in the example of Sect. 5.3, that this reproduce the anomaly discussed in [63].

### ’t Hooft anomaly for the 1-form symmetry

So far we have studied the bulk supergravity action, but the effective 6d anomaly theory can receive contributions from the Chern–Simons terms of the 5-brane sources. To be consistent we add these Chern–Simons actions in the $SL(2,\mathbb {Z})$ covariant form [102, 103], which in general reads,

129 $$\begin{aligned} S^{CS}_{6d} = \sum _{\ell } \int \widetilde{Q}^{\ell I} \sigma _{IJ} \hat{C}^J e^{ Q^{\ell I} \sigma _{IJ} \hat{F}^{J\ell }} \sqrt{\hat{\mathcal {A}}(R_{\mathcal {T}})/\hat{\mathcal {A}}(R_{\mathcal {N}})}, \end{aligned}$$

where the sum over the 5-branes and on each 5-brane we have $\hat{C}=\hat{C}_0 + \hat{C}_2 + \hat{C}_4 + \hat{C}_6 + \hat{C}_8$, $\hat{\mathcal {A}}$ is the A-roof genus of $R_\mathcal {T},R_\mathcal {N}$ tangent and normal bundle curvatures respectively. The expansion in terms of Pontryagin classes reads

130 $$\begin{aligned} \sqrt{\hat{\mathcal {A}}(R_{\mathcal {T}})/\hat{\mathcal {A}}(R_{\mathcal {N}})} = 1 - \frac{1}{48} (p_1(R_{\mathcal {T}})-p_1(R_{\mathcal {N}}))+ \ldots . \end{aligned}$$

Moreover $\widetilde{Q}$ is defined by

131 $$\begin{aligned} \widetilde{Q}= \begin{pmatrix} \tilde{p}_1 &{} \tilde{q}_1 \\ \vdots &{} \vdots \\ \tilde{p}_L &{} \tilde{q}_L \\ \end{pmatrix}, \qquad \widetilde{Q}^{\ell [I} Q^{J],\ell } = \frac{1}{2}\epsilon ^{IJ} \quad \forall \ell . \end{aligned}$$

In addition, we have

132 $$\begin{aligned} \begin{aligned}&\hat{F}^{\ell }= Q^{\ell I} \sigma _{IJ} (d\hat{a}^{\ell J} + \mathcal {C}_2^J)\\&\hat{C}_0 = c_0\\&\hat{C}_2 = \mathcal {C}_2\\&\hat{C}_4 = c_4- \frac{1}{2} Q^{\ell I} \sigma _{IJ} \mathcal {C}_2^J \wedge Q^{\ell I} \sigma _{IJ} \mathcal {C}_2^J\\&\hat{C}_6= \mathcal {C}_6 + c_4 \wedge Q^{\ell I} \sigma _{IJ} \mathcal {C}_2^J+ \frac{1}{6} \widetilde{Q}^{\ell I} \sigma _{IJ} \mathcal {C}_2^J \wedge Q^{\ell I} \sigma _{IJ} \mathcal {C}_2^J \wedge Q^{\ell I} \sigma _{IJ} \mathcal {C}_2^J \\&\hat{C}_6 = \mathcal {C}_8 + \frac{1}{24} (\widetilde{Q}^{\ell I} \sigma _{IJ} \mathcal {C}_2^J) \wedge (Q^{\ell I} \sigma _{IJ} \mathcal {C}_2^J)^3, \end{aligned} \end{aligned}$$

where $\mathcal {C}_6=(b_6,c_6)$, and the $\hat{a}^{\ell I}$ correspond to the center of mass mode of the 5-brane stack and all decouple. We now expand the action and the relevant terms are

133 $$\begin{aligned} S^{CS}_{6d} = \sum _l \int \frac{1}{6} \widetilde{Q} \sigma \mathcal {C}_2 \wedge Q \sigma \mathcal {C}_2 \wedge Q \sigma \mathcal {C}_2 + \frac{1}{48}\widetilde{Q} \sigma \mathcal {C}_2\wedge p_1(R_{\mathcal {T}}) +\ldots , \end{aligned}$$

where we suppressed all the indices in favour of the matrix product, and we ignored the normal bundle contributions. Moreover, there is no contribution of such kind from the bulk (115). In terms of $\widetilde{\mathcal {C}}_2= A^{-1} \sigma \mathcal {C}_2$, which is the diagonal basis where the (*p*, *q*) charges take the Smith normal form. We get the contribution to the SymTFT from the CS couplings to be:

134 $$\begin{aligned} S_{\text {Sym}}^{CS}= \sum _l \int \frac{1}{6} \widetilde{Q} A \widetilde{\mathcal {C}}_2 \wedge Q A \widetilde{\mathcal {C}}_2 \wedge Q A \widetilde{\mathcal {C}}_2 + \frac{1}{48}\widetilde{Q} A \widetilde{\mathcal {C}}_2\wedge p_1(R_{\mathcal {T}}) \end{aligned}$$

with *A* defined such that $Q_{\textrm{SNF}}=BQA$. In particular this action reproduces the cubic anomaly introduced in [58], as we will see in the examples of the next subsection.

### Examples

We now apply these general results to brane-webs that realize 5d SCFTs. This complements the geometric analysis in the earlier parts of the paper, in particular the toric Calabi–Yau reductions in M-theory in Sect. 4. The brane-webs can easily be obtained from the toric diagrams (by passing to a dual graph) and vice versa.

***Example:*** $$SU(2)_0$$ ***SCFT.***

The asymptotic 5-branes charges for the $SU(2)_0$ Seiberg theory are

135 $$\begin{aligned} Q=\begin{pmatrix} 1 &{}1 \\ 1 &{}-1\\ -1&{}-1\\ -1 &{}1 \end{pmatrix} \end{aligned}$$

The Smith normal form reads

136 $$\begin{aligned} Q_{\textrm{SNF}}=\begin{pmatrix} 1 &{} 0\\ 0 &{}2\\ 0 &{}0\\ 0 &{}0\end{pmatrix}, \end{aligned}$$

which together with (118) encodes the $\mathbb {Z}_2$ TQFT that upon a suitable choice of boundary condition determines the $\mathbb {Z}_2$ 1-form symmetry of the theory, together with its background field. We have 2 independent linear combination of the vector fields, and the condition (125) sets one of them to zero. Then we have a contribution to the anomaly coming from (126), and the relevant contribution is

137 $$\begin{aligned} S^{\textrm{bulk}}_{7d} =2 \int d\tilde{a}_1 \wedge \tilde{c}_2 \wedge \tilde{f}_3 + \ldots , \end{aligned}$$

where $d\tilde{a}_1= (\tilde{f}^2_2- \tilde{h}^2_2)$ is the background for the instanton $U(1)_I$ of the gauge theory which enhances to *SO*(3) at the conformal point [63, 83]. $\tilde{c}_2$ is the background field for the $\mathbb {Z}_2$ 1-form symmetry with $\mathbb {Z}/2$ periods, therefore if we map the field to the one with integral periods, $\tilde{c}_2\rightarrow \tilde{c}_2/2$, and integrate on the 6-dimensional boundary we get

138 $$\begin{aligned} S_{\text {Sym}}^{\text {mixed}} =\frac{1}{4} \int d\tilde{a}_1 \wedge \tilde{c}_2\wedge \tilde{c}_2 . \end{aligned}$$

We can also compute the anomaly coming from (134). First of all we use the congruence, that tells us,

139 $$\begin{aligned} \tilde{c}_2 p_1(R_{\mathcal {T}})= 4 \tilde{c}_2 \tilde{c}_2 \tilde{c}_2 \quad \text {mod}\quad 24 \end{aligned}$$

with $\tilde{c}_2$ having integer periods. We also need $\widetilde{Q}$

140 $$\begin{aligned} \widetilde{Q}=\begin{pmatrix} 1+x_1 &{}x_1 \\ -1-x_2 &{}x_2\\ -1+x_3&{}x_3\\ 1-x_4 &{}x_4 \end{pmatrix} , \end{aligned}$$

where $x_i$ are general integer parameters. Evaluating (134) we get,

141 $$\begin{aligned} S_{\text {Sym}}^{\textrm{CS}}=\frac{(x_1+x_4)}{4} \int \tilde{c}_2^3. \end{aligned}$$

where $\tilde{c}_2$ is the one with integer periods. However the freedom of choosing $x_1,x_4$ can be reabsorbed by adding the two terms to the action, which do not change the classical equations of motion of the low-energy anomaly theory,

142 $$\begin{aligned} S^{\textrm{add}}_{7d} = \int (d\tilde{g}_2^2 - 2 \tilde{f}_3) (m \tilde{g}_2^2\wedge \tilde{g}_2^2+m' \frac{1}{48} p_1(R_{\mathcal {T}})) , \end{aligned}$$

where $\tilde{g}_2^2= d\tilde{c}^2_1+ 2 \tilde{c}_2$, moreover $d\tilde{g}_2^2 - 2 \tilde{f}_3$ is no other than of component of the Bianchi identity (127) defining the $\mathbb {Z}_2$ 1-form symmmetry in Stückelberg frame, and $\tilde{g}_2^{\ell } =( B^{-1})^{\ell }_j g_2^j$. Finally, since these are Chern–Simons terms $m, m'$ must be integers. By integrating on the 6d boundary and by plugging in the congruence (139), gauge invariance under $\tilde{c}_2\rightarrow \tilde{c}_2 + 2 d\lambda _1$ fixes $3(x_1+x_4)=4\,m+m'$, which implies

143 $$\begin{aligned} S_{\text {Sym}}^{\text {cubic}}= S_{\text {Sym}}^{\textrm{CS}}+S^{\textrm{add}}_{6d} =0. \end{aligned}$$

***Example:*** $$SU(p)_q$$ ***SCFTs.***

For the general $SU(p)_q$ theory the 5-brane charges and their Smith normal form are

144 $$\begin{aligned} Q=\begin{pmatrix} -p &{}1 \\ q &{}1\\ 0&{}-1\\ (p-q) &{}-1 \end{pmatrix} ,\qquad Q_{\textrm{SNF}}=\begin{pmatrix} 1 &{} 0\\ 0 &{} \text {gcd}(p,q)\\ 0 &{}0\\ 0 &{}0\end{pmatrix} , \end{aligned}$$

which together with (118) encodes the $\mathbb {Z}_{\text {gcd}(p,q)}$ 1-form symmetry of the theory, and its background field. We have 2 independent linear combinations of vector fields, and the condition (125) sets one of them to zero. Then we have a contribution to the anomaly coming from (126) that is,

145 $$\begin{aligned} S^{\textrm{bulk}}_{7d} =-p\int d\tilde{a}_1 \wedge \tilde{c}_2 \wedge \tilde{f}_3 + \ldots , \end{aligned}$$

where $d\tilde{a}_1= ( {\tilde{h}}^2_2)$ generically corresponds to the background for the instanton symmetry $U(1)_I$ of the gauge theory that enhances to *SO*(3) at the superconformal point [83].^16^$\tilde{c}_2$ is the background field for the $\mathbb {Z}_2$ 1-form symmetry with $\mathbb {Z}/\text {gcd}(p,q)$ periods, therefore if we map the field to the one with integral periods, $\tilde{c}_2\rightarrow \tilde{c}_2/\text {gcd}(p,q)$, and integrate on the 6-dimensional boundary we get

146 $$\begin{aligned} S^{\textrm{bulk}}_{6d} =-\frac{p}{2\text {gcd}(p,q)^2} \int d\tilde{a}_1 \wedge \tilde{c}_2\wedge \tilde{c}_2. \end{aligned}$$

This expression is not gauge invariant under $\tilde{c}_2\rightarrow \tilde{c}_2 + \text {gcd}(p,q) d\lambda _1$. We then add the following term which does not change the anomaly theory,

147 $$\begin{aligned} S^{\textrm{add}}_{7d}= n (d\tilde{g}_2^2 - \text {gcd}(p,q) \tilde{f}_3)\wedge d\tilde{a}_1 \wedge \tilde{g}_2^2, \end{aligned}$$

where we recall that $g_2^2= d\tilde{c}^2_1+ \text {gcd}(p,q) \tilde{c}_2$, upon integrating on the 6d boundary, and rescaling $\tilde{c}_2\rightarrow \tilde{c}_2/\text {gcd}(p,q)$, gauge invariance fixes *n* to be an integer such that $\text {gcd}(p,q) n= -p$, such that

148 $$\begin{aligned} S_{\text {Sym}}^{\text {mixed}} =\frac{p(p-1)}{2\text {gcd}(p,q)^2} \int d\tilde{a}_1 \wedge \tilde{c}_2\wedge \tilde{c}_2. \end{aligned}$$

The $\tilde{c}_2^3$ anomaly comes from evaluating (134). First of all we use the congruence, that tells us (139) and we also need $\widetilde{Q}$, that is

149 $$\begin{aligned} \widetilde{Q}=\begin{pmatrix} 1+ p x_1 &{}x_1 \\ 1-px_2 &{}x_2\\ -1&{}x_3\\ -1&{}x_4 \end{pmatrix} , \end{aligned}$$

where $x_i$ are general integer parameters. Evaluating (134) we get

150 $$\begin{aligned} S_{\text {Sym}}^{\textrm{CS}}=\left( \frac{q p}{3 \text {gcd}(p,q)^3}+ \frac{(x_1+x_2)}{4}\right) \int \tilde{c}_2\tilde{c}_2\tilde{c}_2, \end{aligned}$$

where $\tilde{c}_2$ is the one with integer periods. We can now ignore the contribution proportional to $x_1,x_2$. They can be reabsorbed by adding two terms to the action as in the previous example. These new terms do not change the classical equations of motion of the low-energy anomaly theory, since they are proportional to Bianchi identities. They read

151 $$\begin{aligned} S^{\textrm{add}}_{7d} = \int (d\tilde{g}_2^2 - \text {gcd}(p,q) \tilde{f}_3) \wedge \left( m \tilde{g}_2^2\wedge \tilde{g}_2^2+m' \frac{1}{48} p_1(R_{\mathcal {T}})\right) \end{aligned}$$

where $g_2^2= d\tilde{c}^2_1+ \text {gcd}(p,q)\tilde{c}_2$, and $d\tilde{g}_2^2 - \text {gcd}(p,q) \tilde{f}_3$ is no other than of component of the Bianchi identity (127) defining the $\mathbb {Z}_2$ 1-form symmmetry in Stückelberg frame, and $\tilde{g}_2^{\ell } =( B^{-1})^{\ell }_j g_2^j$. By integrating on the 6d boundary and by plugging in the congruence (139), gauge invariance under $\tilde{c}_2\rightarrow \tilde{c}_2+ \text {gcd}(p,q) d\lambda _1$ fixes $m,m'$ to be integers such that $4m+m'= 2(p-3) \frac{p^2 q}{\text {gcd}(p,q)^3}$, which implies

152 $$\begin{aligned} S_{\text {Sym}}= S_{\text {Sym}}^{\text {CS}}+S^{\textrm{add}}_{6d} =\frac{qp(p-2)(p-1)}{6\text {gcd}(p,q)^3} \int \tilde{c}_2\tilde{c}_2\tilde{c}_2. \end{aligned}$$

***Example:*** $$B_N$$ ***SCFTs.***

Finally, we apply this method also to the non-Lagrangian theories of type $B_N$ introduced in Sect. 4 in the toric analysis. The asymptotic 5-branes charges of the web and the SNF are

153 $$\begin{aligned} Q=\begin{pmatrix} N-1 &{}N-2 \\ -1 &{}-(N-1)\\ -(N-2)&{}1\end{pmatrix} ,\qquad Q_{\textrm{SNF}}=\begin{pmatrix} 1 &{} 0\\ 0 &{}N(N-3)+3\\ 0 &{}0\\ 0 &{}0\end{pmatrix} , \end{aligned}$$

which together with (118) encodes the $\mathbb {Z}_{N(N-3)+3}$ 1-form symmetry of the theory, and its background field. The linear relations (111) fix the cycles to be trivial and the pairing as well. So there is no massless vector, corresponding to the theory having no continuous 0-form symmetry. All we can compute is then the cubic anomaly for the 1-form symmetry. To do so we evaluate (134) with the congruence (139), where

154 $$\begin{aligned} \widetilde{Q}=\begin{pmatrix} 1 &{}1 \\ x_1 &{}(N-1)x_1-1\\ -1 - x_2(N-1)&{}x_2\end{pmatrix} , \end{aligned}$$

where $x_1,x_2$ are integer parameters. Plugging this and (139) into (134), we get,

155 $$\begin{aligned} S_{\text {Sym}}^{\text {CS}}=\left( \frac{x_2 (N(N-3)+3) + 2}{6 (N(N-3)+3) } +\frac{x_2}{12} \right) \int \tilde{c}_2 \tilde{c}_2 \tilde{c}_2 . \end{aligned}$$

Again we can add to the supergravity topological action terms which are proportional to Bianchi identities and do not change the classical equations of motion,

156 $$\begin{aligned} S^{\textrm{add}} = \int (d\tilde{g}_2^2 - (N(N-3)+3) \tilde{f}_3) \wedge \left( m \tilde{g}_2^2\wedge \tilde{g}_2^2+m' \frac{1}{48} p_1(R_{\mathcal {T}})\right) . \end{aligned}$$

Again, $m,m'$ are integers that are fixed by gauge invariance under $\tilde{c}_2\rightarrow \tilde{c}_2 + (N(N-3)+3) d\lambda _1$ once we integrated this additional term on a 7d space with a 6d boundary. $m,m'$ then satisfy

157 $$\begin{aligned} 4m+m' = 2 (1-x_2) . \end{aligned}$$

This implies that

158 $$\begin{aligned} S_{\text {Sym}}^{\text {cubic}}= S_{\text {Sym}}^{\textrm{CS}}+S^{\textrm{add}}_{6d} =\frac{(N-2)(N-1)}{6(N(N-3)+3)} \int \tilde{c}_2\tilde{c}_2\tilde{c}_2. \end{aligned}$$

***Example:*** $$B^{(1)}_N$$ ***SCFTs.***

The asymptotic 5-branes charges of the web are

159 $$\begin{aligned} Q=\begin{pmatrix} -1 &{}-(N-1)\\ N-2 &{}N-1 \\ 1 &{} 0\\ -1&{}0\\ -1 &{}0\\ \vdots &{} \vdots \end{pmatrix},\qquad Q_{\textrm{SNF}}=\begin{pmatrix} 1 &{} 0\\ 0 &{}N-1\\ 0 &{}0\\ 0 &{}0\\ \vdots &{} \vdots \end{pmatrix}, \end{aligned}$$

where there are $N-2$ $(-1,0)$ 5-branes. The SNF together with (118) encodes the $\mathbb {Z}_{N-1}$ 1-form symmetry of the theory, and its background field. To compute the cubic anomaly for the 1-form symmetry we evaluate (134) with the congruence (139), where

160 $$\begin{aligned} \widetilde{Q}=\begin{pmatrix} x_1 &{}x_1(N-1)+1 \\ 1&{}1\\ x_2&{}1\\ x_3&{}-1\\ \vdots &{} \vdots \end{pmatrix} , \end{aligned}$$

where $x_1,x_2,x_3, \ldots x_N$ are integer parameters. Plugging this and (139) into (134), we get,

161 $$\begin{aligned} S_{\text {Sym}}^{\text {CS}}=\left( \frac{x_1 (N-1) + 2}{6 (N-1) } +\frac{x_1 +1 }{12} \right) \int \tilde{c}_2\tilde{c}_2 \tilde{c}_2 . \end{aligned}$$

Again we can add to the supergravity topological action terms which are proportional to Bianchi identities and do not change the classical equations of motion,

162 $$\begin{aligned} S^{\textrm{add}} = \int (d\tilde{g}_2^2 - (N-1) \tilde{f}_3) \wedge \left( m \tilde{g}_2^2\wedge \tilde{g}_2^2+m' \frac{1}{48} p_1(R_{\mathcal {T}})\right) . \end{aligned}$$

$m,m'$ are integers that are fixed by gauge invariance under $\tilde{c}_2\rightarrow \tilde{c}_2 + (N-1) d\lambda _1$ once we integrated this additional term on a 7d space with a 6d boundary. $m,m'$ then satisfy

163 $$\begin{aligned} 4m+m' = 2 p -7 -x_1 \end{aligned}$$

This implies that

164 $$\begin{aligned} S_{\text {Sym}}^{\text {cubic}}= S_{\text {Sym}}^{\textrm{CS}}+S^{\textrm{add}}_{6d} =\frac{(N-3)(N-2)}{6(N-1)} \int \tilde{c}_2\tilde{c}_2\tilde{c}_2. \end{aligned}$$

In this example we also have a *U*(1) flavor symmetry. There is indeed a contribution from (126) that is,

165 $$\begin{aligned} S^{\textrm{bulk}}_{7d} =-(N-1)\int d\tilde{a} \wedge \tilde{c}_2 \wedge \tilde{f}_3 + \ldots \end{aligned}$$

where $d\tilde{a}_1= ( \tilde{h}^2_2)$ its the background for the *U*(1) flavor symmetry of the theory, and we display only the interesting contribution. $\tilde{c}_2$ is the background field for the $\mathbb {Z}_2$ 1-form symmetry with $\mathbb {Z}/(N-1)$ periods, therefore if we map the field to the one with integral periods, $\tilde{c}_2\rightarrow \tilde{c}_2/(N-1)$, and integrate on the 6-dimensional boundary we get

166 $$\begin{aligned} S^{\textrm{bulk}}_{6d} =-\frac{1}{2(N-1)} \int d\tilde{a}_1 \wedge \tilde{c}_2\wedge \tilde{c}_2. \end{aligned}$$

This expression is not gauge invariant under $\tilde{c}_2\rightarrow \tilde{c}_2 + (N-1) d\lambda _1$. We then add the following term which does not change the anomaly theory,

167 $$\begin{aligned} S^{\textrm{add}}_{7d}= n \int (d\tilde{g}_2^2 - (N-1)\tilde{f}_3)\wedge d\tilde{a}_1 \wedge \tilde{g}_2^2, \end{aligned}$$

where we recall that $g_2^2= d\tilde{c}^2_1+ (N-1) \tilde{c}_2$, upon integrating on the 6d boundary, and rescaling $\tilde{c}_2\rightarrow \tilde{c}_2/(N-1)$, gauge invariance fixes $n=-1$, such that

168 $$\begin{aligned} S_{\text {Sym}}^{\text {mixed}} =\frac{(N-2)}{2(N-1)} \int d\tilde{a}_1 \wedge \tilde{c}_2\wedge \tilde{c}_2. \end{aligned}$$

***Example:*** $$B^{(2)}_N$$ ***SCFTs.***

The asymptotic 5-branes charges and SNF are

169 $$\begin{aligned} Q=\begin{pmatrix} -1 &{}-N\\ N-1 &{}N \\ -1&{}0\\ -1 &{}0\\ \vdots &{} \vdots \end{pmatrix},\qquad Q_{\textrm{SNF}}=\begin{pmatrix} 1 &{} 0\\ 0 &{}N\\ 0 &{}0\\ 0 &{}0\\ \vdots &{} \vdots \end{pmatrix} , \end{aligned}$$

where there are $N-2$ $(-1,0)$ 5-branes, and the SNF with (118) encodes the $\mathbb {Z}_{N}$ 1-form symmetry of the theory, and its background field. The linear relations (111) fix the cycles to be trivial and the pairing as well. So there is no massless vector, corresponding to the theory having no continuous 0-form symmetry. All we can compute is then the cubic anomaly for the 1-form symmetry. To do so we evaluate (134) with the congruence (139), where

170 $$\begin{aligned} \widetilde{Q}=\begin{pmatrix} x_1 &{}x_1N+1 \\ 1&{}1\\ x_2&{}-1\\ \vdots &{} \vdots \end{pmatrix}, \end{aligned}$$

where $x_1,x_2,x_3, \ldots x_{N-1}$ are integer parameters. Plugging this and (139) into (134), we get

171 $$\begin{aligned} S_{\text {Sym}}^{\text {CS}}=\left( \frac{x_1 N + 2}{6 N } +\frac{x_1}{12} \right) \int \tilde{c}_2\tilde{c}_2 \tilde{c}_2 . \end{aligned}$$

Again we can add to the supergravity topological action terms which are proportional to Bianchi identities and do not change the classical equations of motion,

172 $$\begin{aligned} S^{\textrm{add}} = \int (d\tilde{g}_2^2 - N\tilde{f}_3) \wedge \left( m \tilde{g}_2^2\wedge \tilde{g}_2^2+m' \frac{1}{48} p_1(R_{\mathcal {T}})\right) . \end{aligned}$$

Here $m,m'$ are integers that are fixed by gauge invariance under $\tilde{c}_2\rightarrow \tilde{c}_2 + N d\lambda _1$ once we integrated this additional term on a 7d space with a 6d boundary. $m,m'$ then satisfy

173 $$\begin{aligned} 4m+m' = 2 p -7 -x_1 . \end{aligned}$$

This implies that

174 $$\begin{aligned} S_{\text {Sym}}^{\text {cubic}}= S_{\text {Sym}}^{\text {CS}}+S^{\textrm{add}}_{6d} =\frac{(N-1)(N-2)}{6N} \int \tilde{c}_2\tilde{c}_2\tilde{c}_2 . \end{aligned}$$

We can also compute the ’t Hooft anomaly for the $\mathbb {Z}_N$ 1-form symmetry form symmetry of the $B^{(3)}_N$. The $S_{\text {Sym}}^{\text {CS}}$ has trivial $\frac{1}{N}$ contribution sufficiently implies that $S_{\text {Sym}}^{\text {cubic}}=0$.

These examples illustrate that the approach using the webs and the geometry agree nicely and reproduce the same anomalies also in non-Lagrangian theories. In the context of the webs it is possible to describe theories, which are not described by toric geometries (but so-called generalized toric polygons (GTPs) [104–106]). One can still compute from the web-data using a simple combinatorial prescription the 1-form symmetry [37]. It would be very interesting to generalize the above analysis to webs which are dual to such GTPs.

## Little String Theory Anomaly from Linear Dilaton Holography

The final application is more holographic in spirit, but follows the same philosophy as the other parts of our analysis. We derive he anomaly of the LST from a holoraphic point of view—again using an expansion on the boundary of the space.

*N* NS5 branes in IIB are believed to be described by a Little String Theory [80], that is a non-gravitational theory with non-local stringy excitation [107]. The low-energy limit of this 6d theory is given by (1, 1) SYM in 6 dimensions. Many little string theories have 7d bulk gravity dual solution with linear dilaton behaviour [36]. In this case the dual is given by (string frame)

175 $$\begin{aligned} ds^2= dx_6^2 + N (d \rho ^2 + d\Omega _3), \qquad e^{\phi } = e^{\phi _0 - \rho }, \qquad H= N \text {vol}_3, \end{aligned}$$

where $dx_6^2$ is an abbreviation for a flat 6-dimensional Minkowski metric, $d\Omega _3$ is the metric on a round 3-sphere and $\hbox {vol}_3$ its volume, whereas $\phi _0$ is a constant.

Given this background we can now implement a reduction of the IIB topological coupling

176 $$\begin{aligned} S_{\text {top}}=\int F_5 \wedge H_3 \wedge F_3, \end{aligned}$$

where we expand the fluxes on the cycle of the internal $S^3$ geometry,

177 $$\begin{aligned}&F_5 = f_5 + f_2 \wedge \text {vol}_3\end{aligned}$$

178 $$\begin{aligned}&F_3 = f_3 \end{aligned}$$

179 $$\begin{aligned}&H_3 = h_3 +N \text {vol}_3 . \end{aligned}$$

Because of self-duality of $F_5$, satisfy $f_5= *_7 f_2$, where $*_7$ is the hodge dual in the seven-dimensional space spanned by $\hbox {Minkowski}_6$ and the $\rho$ coordinate. The Bianchi identity $dF_3=dH_3=0$ and $dF_5=H_3\wedge F_3$ imply that

180 $$\begin{aligned} df_3=0&\quad \rightarrow \quad h_3 = db_2 \end{aligned}$$

181 $$\begin{aligned} dh_3 = 0&\quad \rightarrow \quad f_3= dc_2\end{aligned}$$

182 $$\begin{aligned} df_2 = N f_3&\quad \rightarrow \quad f_2 = dc_1 + N c_2 \end{aligned}$$

183 $$\begin{aligned} df_5 = N h_3\wedge f_3&\quad \rightarrow \quad f_5 = dc_4 - N c_2 \wedge db_2. \end{aligned}$$

Since self-duality of $F_5$ imposes $f_5= *_7 f_2$ we manifestly have two frames.

***BF-frame.***

In this frame we choose to keep $f_5$, and therefore what we get is the following coupling,

184 $$\begin{aligned} S_{{\text {top}}_{8d}} = N \int dc_4 \wedge dc_2 - N \int db_2 \wedge c_2 \wedge dc_2 . \end{aligned}$$

The first one give rise to the singleton theory, which upon choice of boundary condition, fixes whether the boundary theory has a $\mathbb {Z}_N$ 1-form symmetry with background $c_2$, such that $Ndc_2=0$, or a dual 3-form symmetry, or a mixture [22–25, 27–30, 108, 109]. The background $c_2$ corresponds to the center 1-form symmetry of the 6d *SU*(*N*) gauge theory low-energy limit of the LST. The second coupling is the holographic realization of part of the mixed anomaly between the continuous 1-form symmetry associated with the current $J=*_6 \text {Tr} (F\wedge F)$ of the low-energy 6d *SU*(*N*) gauge theory [6], whose background is $b_2$, and $c_2$, the background of the discrete $\mathbb {Z}_N$ 1-form symmetry corresponding to the center of the *SU*(*N*), with periods such that $\oint c_2 \in \frac{\mathbb {Z}}{N}$.

***Stückelberg-frame.***

In this frame the discrete $\mathbb {Z}_N$ 1-form symmetry is realised via the Stückelberg mechanism that is induced by the kinetic term for $f_2=dc_1 + N c_2$. This implies that $c_1$ can be entirely gauged away, and $c_2$ is the background for a $\mathbb {Z}_N$ 1-form symmetry. The surviving topological coupling is then,

185 $$\begin{aligned} S_{\text {top}_{8d}} =- \int db_2 \wedge f_2 \wedge dc_2 , \end{aligned}$$

which will give rise to part of the mixed anomaly among the 2 different 1-forms symmetries of the theory. In order to evaluate the full mixed anomaly we need to take into account the invariance under large gauge transformation for the $b_2$,

186 $$\begin{aligned} b_2 \rightarrow b_2 + \Lambda _2, \qquad \oint \Lambda _2 \in \mathbb {Z} \end{aligned}$$

for any integer value of *N*. The expression (185) is not invariant under these large gauge transformation on a space with a non-trivial boundary. Indeed the term,

187 $$\begin{aligned} S_{\text {top}_{7d}} =- \frac{N}{2}\int db_2 \wedge c_2 \wedge c_2 \end{aligned}$$

is not invariant under (186). The general principle is that any effective action describing the reduction of the IIB action on $S^3$ and in particular the correct classical equations of motion, is valid. We can indeed add the following term,

188 $$\begin{aligned} S_{\text {top}_{8d}} =- N \int db_2 \wedge f_2 \wedge dc_2 - m \int db_2 \wedge f_2\wedge (N f_3 - df_2) , \end{aligned}$$

where adding the second term does not change the theory and the classical configuration, since $(N f_3 - df_2)$ is vanishing due to the Bianchi identity (182). We can now pick this action and integrate on a space with a 7d boundary to obtain the SymTFT

189 $$\begin{aligned} S_{\text {Sym}}=- \frac{N}{2} \int db_2 \wedge c_2 \wedge c_2 + m \frac{N^2}{2} \int db_2 \wedge c_2 \wedge c_2. \end{aligned}$$

where we forget about total derivatives. We need to require that this action is gauge invariant under (186) and that the coefficient is well defined modulo 1. This fixes $m=1$, so that the final result for the anomaly reads,

190 $$\begin{aligned} S_{\text {Sym}}= \frac{N-1}{2N} \int db_2 \wedge \tilde{c}_2 \wedge \tilde{c}_2 , \end{aligned}$$

where $$\frac{\tilde{c}_2}{N} = c_2$$.

## Conclusions and Outlook

The main object of study in this paper is the Symmetry Topological Field Theory (SymTFT). This is an object that encodes the choice of symmetries given the local dynamics, and the anomalies of these symmetries.

Our main result is that this SymTFT can be computed from string/M-theory by a reduction on the boundary of the compactifaction spacetime—this can be in a purely geometric engineering setup, as in the M-theory analysis in Sects. 4 and 3, but also brane-setups fall into this framework, as in Sect. 5.

In the geometric engineering setup we made a case that this requires a refined notion of dimensional reduction, recasting the topological terms in the supergravity action in terms of differential cohomology classes. This differential cohomology approach is *essential* in order to accommodate torsion (co)-cycles and their contributions. The latter is pertinent when the symmetries under consideration are discrete, such as is commonly the case in higher-form symmetries.

There are various obvious applications to other geometric engineering setups. For instance, within the M-theory setting that we have discussed in this paper the natural extension is to consider reduction to 4d on $G_2$-holonomy manifolds, such as the ones proposed by Bryant and Salamon [110], and generalizations thereof, which model the confining-deconfining transition of SYM theories. The reduction on Calabi–Yau four- and five-folds should also result in interesting anomalies in 3d and 1d. Specializing to the case of elliptic Calabi–Yau *n*-folds, the results in M-theory have an uplift to F-theory, and thus anomalies in the context of even-dimensional QFTs, like 6d SCFTs (for a review see [111]), 4d $\mathcal {N}=1$ SQFTs (for a review see [112]) and 2d (0,2) theories [113]. More generally, type IIB compactifications can yield supersymmetric gauge theories, which can have 1-form symmetries [22, 114–117].

In addition to the existence of 1-form (and higher-form) symmetries and resulting anomalies, supersymmetric QFTs can have higher-group symmetries. E.g. in 6d SCFTs [118] and LSTs [6, 119], 5d SCFTs [83], and 4d class S [120]. Such higher-groups (continuous or discrete) are dual to mixed anomalies, and thus should have an imprint in the supergravity link reduction. Beyond this multitude of purely geometric constructions there are brane-systems, and of course the more familiar setting of holography. We hope to return to many of these applications in the future.

## Appendix A: Integral Quantization of $G_4$-Flux

In order to diagnose whether $G_4$ has integral or half-integral periods on a four-cycle $\mathcal {C}_4$, we can compute the integral of the fourth Stiefel-Whitney class of the tangent bundle $T \mathcal {M}_{11}$ on $\mathcal {C}_4$ [69],

A1 $$\begin{aligned} \int _{\mathcal {C}_4} G_4 = \frac{1}{2} \, \int _{\mathcal {C}_4} w_4(T\mathcal {M}_{11}) \mod 1 \ , \end{aligned}$$

where the pullback to $\mathcal {C}_4$ is implicit. In this work, we consider 11d spacetimes that are a direct product, $\mathcal {M}_{11} = \mathcal {W}_{11-n} \times L_n$, where $n=3$ and $L_3 = S^3/\Gamma$ (with $\Gamma$ and ADE subgroup of *SU*(2)), or $n=5$ and $L_5$ a smooth Sasaki–Einstein manifold.

The total Stiefel-Whitney class splits as $w(T\mathcal {M}_{11}) = w(T\mathcal {W}_{11-n}) \smile w(T L_n)$. Possible contributions to $w_4(T\mathcal {M}_{11})$ are therefore of the form $w_{4-i}(T\mathcal {W}_{11-n}) \smile w_i(TL_n), i =0,1,2,3,4$. We observe that all the spaces $L_n$ in this work are the base of a Calabi–Yau cone. In particular, each $L_n$ is orientable and Spin, and therefore $w_1(TL_n) = 0 = w_2(TL_n)$. We also assume that external spacetime is orientable and Spin, so that $w_1(T\mathcal {W}_{11-d}) = 0 = w_2(T\mathcal {W}_{11-n})$. We conclude that terms of the form $w_{4-i}(T\mathcal {W}_{11-n}) \smile w_i(TL_n)$ with $i=1,2,3$ cannot give any non-zero contributions to $w_4(T\mathcal {M}_{11})$. The remaining potential contributions thus have $i=0$ or $i = 4$. To kill the contribution with $i=0$ we assume that external spacetime satisfies $w_4(T\mathcal {W}_{11-n}) = 0$. What remains to be checked is whether $w_4(TL_n)$ is trivial. Clearly, we have $w_4(TL_3) = 0$ for dimensional reasons.^17^ Next, we prove that $w_4(TL_5) =0$ for any smooth Sasaki–Einstein five-manifold $L_5$. In fact, we can prove a stronger statement: $w_{i>0}(TL_5) = 0$.

Let us adopt the shorthand notation $w_i= w_i(TL_5)$. We have already observed $w_1 = 0 = w_2$. We also have (in general, from the Wu formula)

A2 $$\begin{aligned} \textrm{Sq}^{1}(w_2) = w_3 + w_1\smile w_2 \ , \end{aligned}$$

which implies $w_3=\textrm{Sq}^{1}(0)=0$ in our case. We also have that the Wu class

A3 $$\begin{aligned} \nu _4 = w_1^4 + w_2^2 + w_1\smile w_3 + w_4 \end{aligned}$$

necessarily vanishes on a 5-manifold for degree reasons (as it represents $\textrm{Sq}^{4}$ acting on $H^1(L_5;\mathbb {Z}_2)$, which vanishes by general properties of the Steenrod squares), so we conclude $w_4=0$. Finally, again from the Wu formula

A4 $$\begin{aligned} \textrm{Sq}^{2}(w_3) = w_2\smile w_3 + w_1\smile w_4 + w_5 \ , \end{aligned}$$

which implies, given $w_1=w_2=w_3=0$, that $w_5=0$.

Let us conclude with further comments on $p_1 = p_1(TL_5)$. Note that on a Spin manifold we have [121]

A5 $$\begin{aligned} \mathfrak {P}(w_2) = \rho _4(p_1) + \theta _2(w_1\textrm{Sq}^{1}(w_2) + w_4) \ , \end{aligned}$$

so, since $w_1=w_2=w_4=0$, we learn from the analysis above that $\rho _4(p_1)=0$, or equivalently that $p_1$ vanishes mod 4.

## Appendix B: Type IIA Analysis

We have seen that the differential cohomological approach is very effective in computing the SymTFTs starting from M-theory. To cross-check and to complement this with more familiar methods in this appendix we compute the same SymTFTs using the IIA background, which is given by not only geometry but also RR-flux. This setups is however more canonical in that there are no torsion cycles. The results will agree (to the extent that the more limited IIA analysis below is applicable, not every M-theory background that we analyse above has a well-behaved IIA limit), but this more conventional approach will be a countercheck and contrast to the much more efficient differential cohomology approach that we are proposing.

### B.1 Type IIA background for 7d SYM

The Einstein metric on $S^3/{\mathbb {Z}}_N$ that descends from the quotient of the round unit-radius metric on $S^3$ can be written as

B6 $$\begin{aligned} ds^2(S^3/{\mathbb {Z}}_N) = \frac{1}{4} \, (d\theta ^2 + \sin ^2 \theta \, d\phi ^2) + \frac{1}{N^2} \, D\psi ^2 \ , \qquad D\psi = d\psi - \frac{N}{2} \cos \theta \, d\phi . \end{aligned}$$

The coordinate $\theta$ has range $[0,\pi ]$ and the angles $\phi$, $\psi$ have period $2\pi$. We identify the Hopf fiber $S^1_\psi$ in (B6) with the M-theory circle. It then follows that in type IIA the link geometry consists simply of a round $S^2$ (described by the coordinates $\theta , \phi$ in (B6)) threaded by *N* units of $F_2$ R-R 2-form flux. Let us stress that in the type IIA setup there are no torsional cohomology classes. As a result, the reduction can be performed according to the standard paradigm, including only the modes associated to harmonic forms in the internal space. In the present situation, the latter is $S^2$ and the only non-trivial harmonic forms are 1 and

B7 $$\begin{aligned} \omega _2 = \frac{1}{4\pi } \, \sin \theta \, d\theta \wedge d\phi \ , \qquad \int _{S^2} \omega _2 = 1 . \end{aligned}$$

The Chern–Simons topological terms in the Type IIA effective action can be written as an 11-form

B8 $$\begin{aligned} S_{\textrm{10d,top}} = 2\pi \int _{\mathcal {M}_{10}} I_{10}^{(0)} \ , \qquad dI_{10}^{(0)} = I_{11} = - \frac{1}{2} \, {H_3}\wedge {F_4} \wedge {F_4}- {H_3} \wedge X_8 . \end{aligned}$$

The 8-form $X_8$ is as in M-theory, but constructed with Pontryagin classes of the tangent bundle to 10d spacetime. The 3-form $H_3$ is the field strength of the NS-NS 2-form field, while $F_4$ is the field strength of the R-R 3-form field. We will not consider in this section $X_8$. The Bianchi identities for $H_3$, $F_4$ read^18^

B9 $$\begin{aligned} dH_3 = 0 \ , \qquad {d F_4} = - {H_3} \wedge {F_2} \ , \end{aligned}$$

where $F_2$ is the field strength of the RR 1-form field, obeying $dF_2 = 0$.

As already noted above, we have a non-zero $F_2$ flux threading $S^2$

B10 $$\begin{aligned} {F_2 }= N \, \omega _2 . \end{aligned}$$

The expansions of the field strengths $H_3, F_4$ take the form

B11 $$\begin{aligned} {H_3} = { {\widetilde{H}}_3} + { f_1} \wedge \omega _2 \ , \qquad {F_4} = - {\gamma _4} - {{\widetilde{F}}_2} \wedge \omega _2 . \end{aligned}$$

The quantities ${\widetilde{H}}_3, f_1, \gamma _4, {\widetilde{F}}_2$ are external field strengths, whose Bianchi identities follow from (B9), (B10),

B12 $$\begin{aligned} d{\widetilde{H}}_3= 0 \ , \qquad df_1 = 0 \ , \qquad d\gamma _4 = 0 \ , \qquad d{\widetilde{F}}_2 = N \, {\widetilde{H}}_3 . \end{aligned}$$

Upon plugging (B11) into $I_{11}$ in (B8) and fiber integrating along $S^2$, we obtain the following 9-form expression in the external field strengths,

B13 $$\begin{aligned} I_9 = - { {\widetilde{F}}_2 } \wedge {{\widetilde{H}}_3} \wedge {\gamma _4} . \end{aligned}$$

The key subtlety in the following is to note that we have the freedom of adding an integer multiple of the Bianchi identity to this anomaly

B14 $$\begin{aligned} I_9^{\text {extra}} = (N \widetilde{H}_3 - d \widetilde{F}_2 ) \widetilde{F}_2 \gamma _4 = N \widetilde{H}_3 \widetilde{F}_2 \gamma _4 - d(\widetilde{F}_2 \widetilde{F}_2 \gamma _4) . \end{aligned}$$

Adding these contributions

B15 $$\begin{aligned} I_9^{\text {improved}} =( N-1) \widetilde{H}_3 \widetilde{F}_2 \gamma _4 - d(\widetilde{F}_2 \widetilde{F}_2 \gamma _4) \end{aligned}$$

Applying an anti-derivative, and dropping total derivatives, we get

B16 $$\begin{aligned} I_8^{(0)} = {1\over 2} N(N-1) \tilde{F}_2 \tilde{F}_2 \gamma _4 = {(N-1)\over 2 N } B_2 B_2 \gamma _4 , \end{aligned}$$

where $\tilde{F}_2 = B_2/N$, which agrees with the result in the main text. We should note that this analysis, though based on a more standard reduction process, is very limited to situations, where there is a IIA reduction of the M-theory background.

### B.2 Mixed anomaly for M-theory on $C(Y^{p,0})$ from type IIA

Like in the 7d case we can also repeat the computation of the SymTFT from a IIA point of view for the $SU(p)_0$ SCFTs in 5d. Again the circle reduction from M-theory to IIA implies a background flux in the geometry. We take the relation between the 11d supergravity fields and the Type IIA fields (in the string frame)

B17 $$\begin{aligned} ds^2_{11}&= e^{- \frac{2}{3} \, \Phi } \, ds^2_{10} + e^{\frac{4}{3} \, \Phi } \, (dy + C_1)^2 \ , \qquad y \sim y + 2 \pi R \ , \nonumber \\ C_3^{\textrm{11d}}&= C_3 + B_2 \wedge dy . \end{aligned}$$

These relations are written in the supergravity normalization. We work with integral fluxes

B18 $$\begin{aligned} d {\widetilde{F}}_4 = - H_3 \, F_2 \ , \qquad {\mathcal {I}}_{11} = - \frac{1}{2} \, H_3 \, {\widetilde{F}}_4 \, {\widetilde{F}}_4 . \end{aligned}$$

We reduce of $X_4 = S^2 \times S^2$. The volume forms on the two $S^2$ factors are denoted $\omega _{2,a=1}, \omega _{2,a=2}$. The intersection pairing in this basis is

B19 $$\begin{aligned} I_{ab} =\int _{X_4} \omega _{2a} \, \omega _{2b} = \begin{pmatrix} 0 &{} 1 \\ 1 &{} 0 \end{pmatrix} . \end{aligned}$$

The IIA field strengths are given by

B20 $$\begin{aligned} F_2= n^a \, \omega _{2a} \ , \qquad H_3 = {\widetilde{H}}_3 + f_1^a \, \omega _{2a} \ , \qquad {\widetilde{F}}_4 = {\widetilde{u}}_0 \, V_4 + F_2^a \, \omega _{2a} + \gamma _4 . \end{aligned}$$

We have $\int _{X_4} V_4 = 1$. The background flux quanta $n^a$ in the basis used so far are

B21 $$\begin{aligned} n^a = \begin{pmatrix} p \\ p \end{pmatrix} . \end{aligned}$$

The non-trivial Bianchi identities after reduction on $X_4$ are

B22 $$\begin{aligned} dF_2^a = - n^a \, {\widetilde{H}}^3 \ , \qquad d{\widetilde{u}}_0 = - I_{ab} \, n^a \, f_1^b . \end{aligned}$$

We compute

B23 $$\begin{aligned} I_7 = - \int _{X_4} {\mathcal {I}}_{11} = {\widetilde{u}}_0 \, {\widetilde{H}}_3 \, \gamma _4 + I_{ab} \, F_2^a \, f_1^b \, \gamma _4 + \frac{1}{2} \, I_{ab} \, F_2^a \, F_2^b \, {\widetilde{H}}_3 . \end{aligned}$$

Note that we have

B24 $$\begin{aligned} d F_2 ^{a} = - p \tilde{H}^3 \end{aligned}$$

however there is one combination that is a massless *U*(1) gauge field

B25 $$\begin{aligned} F_{I} = F_2^1 - F_2^2 , \end{aligned}$$

and another that is a torsion *p*-form $F_2^1$. We perform this change of basis, which is a lattice automorphism with determinant $+1$, where

B26 $$\begin{aligned} M^a{}_b = \begin{pmatrix} 1 &{} - 1 \\ 1 &{} 0 \end{pmatrix} ,\qquad n'^a = \begin{pmatrix} 0 \\ p \end{pmatrix} \ , \qquad I'_{ab} = \begin{pmatrix} 0 &{} -1 \\ -1 &{} 2 \end{pmatrix} . \end{aligned}$$

In this new basis, we can write

B27 $$\begin{aligned} I_7 = - \int _{X_4} {\mathcal {I}}_{11} = {\widetilde{u}}_0 \, {\widetilde{H}}_3 \, \gamma _4 + I'_{ab} \, (F_{I}, F_2^1)^a \, f_1' {}^b \, \gamma _4 - F_{I} F_2^1 \widetilde{H}_3 + F_2^1 F_2^1 \widetilde{H}_3 , \end{aligned}$$

with the non-trivial Bianchi identities

B28 $$\begin{aligned} d F_2^1 = - p \, {\widetilde{H}}_3 \ , \qquad d{\widetilde{u}}_0 = p \, f_1'^1 - 2 \, p \, f_1'^2 . \end{aligned}$$

We introduce gauge potentials $B_2, c_3$ for ${\widetilde{H}}_3$, $\gamma _4$, respectively,

B29 $$\begin{aligned} {\widetilde{H}}_3 = d\tilde{B}_2 \ , \qquad \gamma _4 = dc_3 . \end{aligned}$$

Let us focus on the case with $\gamma _4=f_1=0$. Again as in 7d there is an ambiguity that we can add a multiple of the Bianchi identity

B30 $$\begin{aligned} I_7^{\text {improved}} = - F_{I} F_2^1 \widetilde{H}_3 + F_2^1 F_2^1 \widetilde{H}_3 + m (d F_2^1 + p \widetilde{H}_3) F_2^1 F_2^1 + n (d F_2^1 + p \widetilde{H}_3) F_2^1 F_I \end{aligned}$$

An antiderivative of $I_7$ again rescaling $\tilde{B}_2 = B_2/p$ we find

B31 $$\begin{aligned} I_6^{(0)} =-{ (1+ m p) \over 3 p^2} B_2^3 - { np-1 \over 2 p} B_2^2 F_I. \end{aligned}$$

We are free to choose *n* to make the term well-defined under gauge transformations. The mixed anomaly between the instanton $U(1)_I$ and the 1-form symmetry is invariant for $n=1$

B32 $$\begin{aligned} I_6^{0-1} = - {p-1 \over 2p} B_2^2 F_I. \end{aligned}$$

Similarly the $B^3$ anomaly has an ambiguity, however as we know from the M-theory computation this also acquires contributions from $C_3 X_8$. The match with the M-theory computation is more subtle and might require a detailed analysis of the higher derivative couplings studied in [122]. We leave this for the future and focus our endeavours on the differential cohomology approach.

## Footnotes

1 In this paper we will be considering cases with $d\in 2\mathbb {Z}+1$, so there are no local anomalies.

2 One complication in the general case is that topological terms in the parent string theory are not the only source of topological terms in the lower dimensional theory. For instance, in the presence of chiral fermions the lower dimensional theory will generically include couplings proportional to the $\eta$ invariant evaluated in the compactification space.

3 In supergravity theories different prescriptions to incorporate torsion have been put forward [38, 39], but none that are mathematically entirely satisfactory or unambiguous—we will comment on a detailed comparison to these approaches shortly.

4 The cases discussed do not have discriminant components of the elliptic fibration intersecting the boundary of the Calabi–Yau.

5 Our understanding is that the results in [57] differ because their choice of non-compact divisor dual to the instanton generator is not always the same as in the field theory, but can differ from that by torsion generators.

6 Using the fact that $\star , \wedge , \smile$ are graded commutative, these identities can also be written in the form

$$\begin{aligned} \breve{b} \star \tau ([\omega ]) = (-1)^q \, \tau ( [ R(\breve{b}) \wedge \omega ] ) \ , \qquad \breve{b} \star i(u) = (-1)^q \, i(I(\breve{b}) \smile u) \ , \end{aligned}$$

for $$\breve{b} \in \breve{H}^q(\mathcal {M}), \omega \in \Omega ^{p-1}(\mathcal {M})$$, $$u\in H^{p-1}(\mathcal {M}; {\mathbb {R}} /{\mathbb {Z}})$$.

7 It should be noted that in the mathematical literature this element of $\breve{H}^4(\mathcal {M}_{11})$ is sometimes denoted $\breve{C}_3$. We prefer the notation $\breve{G}_4$ to make manifest the degree of this differential cohomology class.

8 More subtle questions require working on some *generalised* differential cohomology theory, of which there are many types. The generalised cohomology theory appropriate for M-theory has been postulated in [68]. It would be interesting to see if this more refined picture leads to any interesting consequences in field theory.

9 We refer the reader to [70] for a model for the M-theory 3-form in terms of a shifted differential cohomology class, which can accommodate both integral and half-integral periods.

10 We are implicitly considering a Wick-rotated version of the theory, and we are taking external spacetime $\mathcal {W}_{11-n}$ to be a closed, connected, oriented manifold.

11 Recall that the linking pairing is defined modulo 1, so in all cases—except $D_{2n}$, where $\ell$ is not defined—$\ell$ can be taken to be an integer.

12 Note that there is a genuine ambiguity here: if *t* is a generator of $\mathbb {Z}_n$, so is *kt* for any *k* such that $\gcd (k,n)=1$. We have $CS[S^3/\Gamma ,k\breve{t}_2]=k^2CS[S^3/\Gamma ,\breve{t}_2]$ mod 1, so this rescaling can potentially change the coefficient of the anomaly. This is why it is important to choose the right generator of the torsional group when comparing with field theory results, even though any choice of *k* is in principle physically allowed.

13 We are abusing notation slightly here, and denoting by $B_2$ both the continuous field that we use in writing the BF action and the discrete background for the 1-form symmetry, since they are identified at low energies.

14 The Whitney sum formula for Pontryagin classes in integral cohomology is [93]

83 $$\begin{aligned} p_q(A \oplus B) = \sum r_{2q-j}(A) \smile r_j(B) \ , \quad r_{2s} = p_s \ , \quad r_{2s+1} = \textrm{Bock}(w_{2s}) \smile \textrm{Bock}(w_{2s}) + p_s \smile \textrm{Bock}(w_1) \ , \end{aligned}$$

where *A*, *B* are *O* bundles, the $w_i$’s are Stiefel-Whitney classes, and Bock is the Bockstein homomorphism associated to the short exact sequence $0 \rightarrow {\mathbb {Z}} \xrightarrow {2} {\mathbb {Z}} \rightarrow {\mathbb {Z}}_2 \rightarrow 0$. In Appendix A we show $w_1(TL_5)=w_2(TL_5)=0$, which implies that (83) holds at the level of cohomology with integral coefficients.

15 Globally the 1-cycles can be thought as segments connecting the puctures, such that the constraints (111) are satisfied. F.A. would like to thank Oren Bergman and Christoph Uhlemann for clarifying some of these aspects.

16 We recall that here we have not activated the Stiefel-Whitney class $w_2(SO(3))$ for the non-abelian flavor symmetry. It will be also interesting to compute the mixed anomaly in terms of $w_2$ from a string theory engineering perspective.
